# Extensive Ethnic Variation and Linkage Disequilibrium at the *FCGR2/3* Locus: Different Genetic Associations Revealed in Kawasaki Disease

**DOI:** 10.3389/fimmu.2019.00185

**Published:** 2019-03-21

**Authors:** Sietse Q. Nagelkerke, Carline E. Tacke, Willemijn B. Breunis, Michael W. T. Tanck, Judy Geissler, Eileen Png, Long T. Hoang, Joris van der Heijden, Ahmad N. M. Naim, Rae S. M. Yeung, Michael L. Levin, Victoria J. Wright, David P. Burgner, Anne-Louise Ponsonby, Justine A. Ellis, Rolando Cimaz, Chisato Shimizu, Jane C. Burns, Karin Fijnvandraat, C. Ellen van der Schoot, Timo K. van den Berg, Martin de Boer, Sonia Davila, Martin L. Hibberd, Taco W. Kuijpers, The International Kawasaki Disease Genetics Consortium, Nagib Dahdah

**Affiliations:** Division of Pediatric Cardiology, Sainte-Justine University Hospital Center, University of Montreal, Montreal, Quebec, Canada; Isabelle Kone-Paut, Pediatric Rheumatology and CEREMAIA, Le Kremlin-Bicêtre University Hospital, APHP, Paris-Sud University, Paris, France; ^1^Department of Blood Cell Research, Sanquin Research and Landsteiner Laboratory, Amsterdam UMC, University of Amsterdam, Amsterdam, Netherlands; ^2^Pediatric Hematology, Immunology and Infectious Diseases, Emma Children's Hospital, Amsterdam UMC, University of Amsterdam, Amsterdam, Netherlands; ^3^Department of Clinical Epidemiology, Biostatistics and Bioinformatics, Amsterdam UMC, University of Amsterdam, Amsterdam, Netherlands; ^4^Infectious Diseases, Genome Institute of Singapore, Singapore, Singapore; ^5^Division of Rheumatology, Department of Pediatrics, The Hospital for Sick Children, University of Toronto, Toronto, ON, Canada; ^6^Department of Pediatrics, Imperial College London, London, United Kingdom; ^7^Murdoch Children's Research Institute, Royal Children's Hospital, Melbourne, VIC, Australia; ^8^Department of Paediatrics, University of Melbourne, Melbourne, VIC, Australia; ^9^Faculty of Health, Centre for Social and Early Emotional Development, Deakin University, Burwood, VIC, Australia; ^10^Rheumatology Unit, Meyer Children's Hospital, University of Florence, Florence, Italy; ^11^Department of Pediatrics, University of California San Diego School of Medicine, La Jolla, CA, United States; ^12^Department of Plasma Proteins, Sanquin Research, Amsterdam UMC, University of Amsterdam, Amsterdam, Netherlands; ^13^Department of Molecular Cell Biology and Immunology, Amsterdam Infection and Immunity Institute, Amsterdam UMC, Vrije Universiteit Amsterdam, Amsterdam, Netherlands; ^14^Human Genetics, Genome Institute of Singapore, Singapore, Singapore; ^15^Department of Pathogen Biology, London School of Hygiene and Tropical Medicine, London, United Kingdom

**Keywords:** Fc-gamma receptor, FCGR polymorphism, linkage disequilibrium, Kawasaki disease (KD), immunogenetics

## Abstract

The human Fc-gamma receptors (FcγRs) link adaptive and innate immunity by binding immunoglobulin G (IgG). All human low-affinity FcγRs are encoded by the *FCGR2/3* locus containing functional single nucleotide polymorphisms (SNPs) and gene copy number variants. This locus is notoriously difficult to genotype and high-throughput methods commonly used focus on only a few SNPs. We performed multiplex ligation-dependent probe amplification for all relevant genetic variations at the *FCGR2/3* locus in >4,000 individuals to define linkage disequilibrium (LD) and allele frequencies in different populations. Strong LD and extensive ethnic variation in allele frequencies was found across the locus. LD was strongest for the *FCGR2C*-ORF haplotype (rs759550223+rs76277413), which leads to expression of FcγRIIc. In Europeans, the *FCGR2C*-ORF haplotype showed strong LD with, among others, rs201218628 (*FCGR2A*-Q27W, *r*^2^ = 0.63). LD between these two variants was weaker (*r*^2^ = 0.17) in Africans, whereas the *FCGR2C*-ORF haplotype was nearly absent in Asians (minor allele frequency <0.005%). The *FCGR2C*-ORF haplotype and rs1801274 (*FCGR2A*-H131R) were in weak LD (*r*^2^ = 0.08) in Europeans. We evaluated the importance of ethnic variation and LD in Kawasaki Disease (KD), an acute vasculitis in children with increased incidence in Asians. An association of rs1801274 with KD was previously shown in ethnically diverse genome-wide association studies. Now, we show in 1,028 European KD patients that the *FCGR2C*-ORF haplotype, although nearly absent in Asians, was more strongly associated with susceptibility to KD than rs1801274 in Europeans. Our data illustrate the importance of interpreting findings of association studies concerning the *FCGR2/3* locus with knowledge of LD and ethnic variation.

## Introduction

The human cellular receptors for Immunoglobulin G (IgG), the Fc-gamma receptors (FcγR), have an important role in immunity by linking the adaptive and innate immune systems. Many genetic variations in the genes encoding FcγRs have been found to be associated with auto-immune (1–5), auto-inflammatory (6–8), and infectious diseases (9, 10), and with efficacy of immunotherapy in cancer patients (11–15). Several activating and one single inhibitory FcγR (FcγRIIb) exist, with differential expression on various leukocyte subsets (16, 17). Human FcγRs can be distinguished into one high-affinity receptor (FcγRI) and five low-affinity FcγRs (the different isoforms of FcγRII and FcγRIII) (16, 17). All five genes encoding the low-affinity FcγRs (*FCGR2A, FCGR2B, FCGR2C, FCGR3A*, and *FCGR3B)* are located in a complex gene cluster at chromosome 1q23.3. Many functionally relevant single nucleotide polymorphisms (SNPs) and copy number variants (CNVs) are described in the *FCGR2/3* locus, leading to altered receptor functions ranging from different binding affinity to IgG to complete absence of expression of certain genes (17–19). The *FCGR2/3* locus involves a segmental duplication, making it constitutively difficult to genotype because of the high degree of homology between the genes (18, 20). Due to the close proximity of all the five different *FCGR2* and *FCGR3* genes, the polymorphic variants in these genes are likely to be in strong Linkage Disequilibrium (LD). However, except for some incidental reports on LD between some of the SNPs (21–24), a comprehensive analysis of LD between the functional variants at this locus has not been previously performed.

One of the diseases in which only one genetic variant of the *FCGR2/3* locus has been thoroughly studied is Kawasaki Disease (KD). KD is an acute systemic vasculitis that predominantly occurs in children <5 years (25). About 25% of untreated KD patients develop coronary artery aneurysms, which may lead to ischemic heart disease, myocardial infarction and sudden death at young age (26). Although the etiology of KD remains unknown, the general consensus is that KD reflects an abnormal inflammatory response to an unknown infectious trigger in genetically susceptible individuals. Standard treatment consists of a single infusion of high-dose intravenous immunoglobulins (IVIg) in combination with aspirin (27). Although the mechanism of action of IVIg in KD is unclear, early treatment shortens the duration of fever and reduces the incidence of coronary artery aneurysms to less than 5% (28). Since IVIg therapy is effective in the majority of patients, the receptors for IgG, the Fc-gamma Receptors (FcγRs), are of particular interest in KD research.

In our GWAS study on KD (6), we identified the *FCGR2A*-131H SNP (rs1801274) to be associated at genome-wide significance. This variant results in a substantial difference in the ability of FcγRIIa to bind the human IgG2 subclass (19). rs1801274 shows the strongest evidence of association with KD and this finding has been intensively studied and validated in a number of cohorts of varying ethnicity (6, 7, 29–34). Apart from the *FCGR2A*-H131R SNP (rs1801274), only a few other SNPs in this locus have been evaluated for KD susceptibility, without any significant association (29–31). Nevertheless, because of the sequence homology and the genetic complexity, a very large part of the *FCGR2/3* locus was not covered in GWAS or other studies before. Hence, we postulated that other variants at the locus may also play a role in KD susceptibility, which could either be tagged by *FCGR2A*-131H (rs1801274), or act independently. To address this, we performed further fine-mapping of the *FCGR2/3* gene cluster in a case-control as well as a family-based linkage study with a total of 1,028 patients with KD, and genotyped healthy control individuals of different ethnic groups to define LD and ethnic variation. We used a previously developed accurate multiplex ligation-dependent probe amplification (MLPA) assay covering all the functionally relevant SNPs and CNVs at the *FCGR2/3* locus (5).

In the present study, including more than 4,000 individuals, we found marked ethnic differences in allele frequencies for most of the SNPs and CNVs. The most prominent difference was observed for the *FCGR2C*-ORF haplotype, which we have previously shown to result in expression of the activating FcγRIIc (35). In most individuals, FcγRIIc cannot be expressed as a result of a polymorphic stop codon in exon3 (rs759550223), but the expressed *FCGR2C*-ORF haplotype is associated with susceptibility to immune thrombocytopenic purpura (5). We now show that the *FCGR2C*-ORF haplotype is virtually absent in Asian and African populations. *FCGR2C*-ORF is in very strong LD with several other SNPs in the European population, but could be identified as a novel susceptibility haplotype for KD in this population, independent of the *FCGR2A*-H131R SNP. Our comprehensive analysis of the *FCGR2/3* locus will greatly contribute to a better understanding of the relevance of the different FcγRs in inflammatory diseases.

## Subjects and Methods

### Study Populations

#### KD Cases

Unrelated KD cases were recruited from Australia, The Netherlands and the United States. All cases from Australia (109) and the United States (62) were also included in our previous GWAS (6), whereas the cases from the Netherlands (234) consisted of 166 cases from the GWAS and 68 new cases. There was no overlap with patients in the study previously reported by Biezeveld et al (30). The diagnosis of KD was based on the standard diagnostic clinical criteria from the American Heart Association.

#### Cohorts of Control Subjects

##### Europeans

Since no DNA of the control population in our previous GWAS was available, we genotyped a new group of unrelated controls of European descent, consisting of healthy individuals from Austria (478), Australia (156), The Netherlands (199), and the United Kingdom (86). All were of European descent by self-reported ethnicity (36, 37).

##### Chinese

The Chinese population consisted of 428 healthy individuals from Canada of Han-Chinese descent, all of which were grandparent-proven Han-Chinese.

##### African

The South African population consisted of 149 healthy blood donors of African descent by self-reported ethnicity as reported before (38). The Ethiopian population consisted of 142 healthy blood donors of African Ethiopian descent by self-reported ethnicity (38). The West African population consisted of 65 sickle-cell disease patients from the Netherlands, all of which were of West-African descent by self-reported ethnicity, including individuals from Ghana (52), Nigeria (4), Sierra Leone (4), Togo (3), and Cameroon (2). The Surinam population consisted of 78 sickle-cell disease patients of African Surinamese descent by self-reported ethnicity. The Antillean population consisted of 6 sickle-cell disease patients from the Netherlands who were from Curaçao and were of African Caribbean descent by self-reported ethnicity, and 68 healthy blood donors from Curaçao who were of African Caribbean descent by self-reported ethnicity as described previously (38).

##### Family-based association study

623 KD patients (none overlapping with the case control study) were included, consisting of KD patients from the United States (386, of which 348 complete trios and 38 incomplete trios, 153 European), Australia (104, all complete trios, 72 European) and the Netherlands (98, all complete trios, 82 European) and Italy (35, all complete trios, all Mediterranean). All KD patients in the family-based association study from the United States and Australia were included in our previous GWAS (6), the patients from the Netherlands and Italy were new.

In total, 4,091 individuals were genotyped. Table S1 provides an overview of all individuals. This study was carried out in accordance with the recommendations of the Kawasaki Study Protocol approved by the Medical Ethical Committee at the Academic Medical Centre in Amsterdam, the Netherlands, with written informed consent from all subjects. All subjects gave written informed consent in accordance with the Declaration of Helsinki. The protocol was approved by the Medical Ethical Committee at the Academic Medical Centre in Amsterdam, the Netherlands and by the medical ethical committees of the other participating centers.

### Clinical Data

Clinical information was collected by review of the clinical KD registries. CAAs were defined based on the definition of the Japanese Ministry of Health or Z-scores >2.5 according to the Boston Z-score data. According to the definition of the Japanese Ministry of Health a coronary artery was considered abnormal if the diameter of the internal lumen was > in children younger than 5 years or > in a child aged 5 years or older, or if the internal diameter of a segment was at least 1.5 times larger than that of an adjacent segment. IVIg response was determined in the patients receiving treatment with IVIg within 11 days after the disease onset. Patients who received more than one dose of IVIg because of persistent or recrudescent fever more than 36 h after the initial IVIg dose were defined as IVIg non-responders.

### Genotyping by MLPA and Construction of Haplotypes From MLPA Data

The MLPA assay was performed according to the manufacturer's protocol, essentially as described previously (5, 39) and is described in great detail in the Supplemental Methods.

### Flow Cytometry, Gene Expression Microarray and RT-qPCR

Flow cytometry, gene expression microarray and RT-qPCR were performed as described in the Supplemental Methods.

### Statistical Analysis

#### Genotype/Allele Frequencies and Linkage Disequilibrium

Differences in copy number and allele frequencies between (sub)populations and differences in allele frequencies between groups of individuals with normal, decreased and increased copy number were tested using Fisher's Exact test. Haplotype frequencies and linkage disequilibrium (expressed as *r*^2^ or D') between (multiallelic) markers were estimated in the populations and the parents from the KD trios using the gap package (40) (version 1.1-12).

#### Association With Susceptibility to Kawasaki Disease (KD)

In the case-control study, genotype frequencies were compared between KD cases and healthy controls using Fisher's exact test and odds ratios were estimated using (multiple) logistic regression. In the parent-affected offspring trios, the association between KD and the markers was examined using the (multimarker) FBAT (TDT) test statistic from the FBAT toolkit (41). Results from the case-control and KD trios were meta-analyzed using a fixed effect model and the generic inverse variance method following an approach described by Kazeem and Farrall (42) and using Review Manager software (Version 5, Cochrane Collaboration).

#### Comparison of Expression Levels

In case of multiple expression values per donor, the mean of these values was taken for the statistical analyses. Expressions between groups were compared using Mann-Whitney tests (two groups) or a Kruskal-Wallis test with *post-hoc* Mann-Whitney tests (>2 groups) using GraphPad Prism 6.02.

Apart from the TDT and meta-analyses and the expression analysis, all statistical analyses were carried out using R software (Version 3.0.3, R Core Team). A *p*-value below 0.05 was considered as statistically significant.

## Results

### Characterization of the *FCGR2/3* Locus

**Figure 1:**
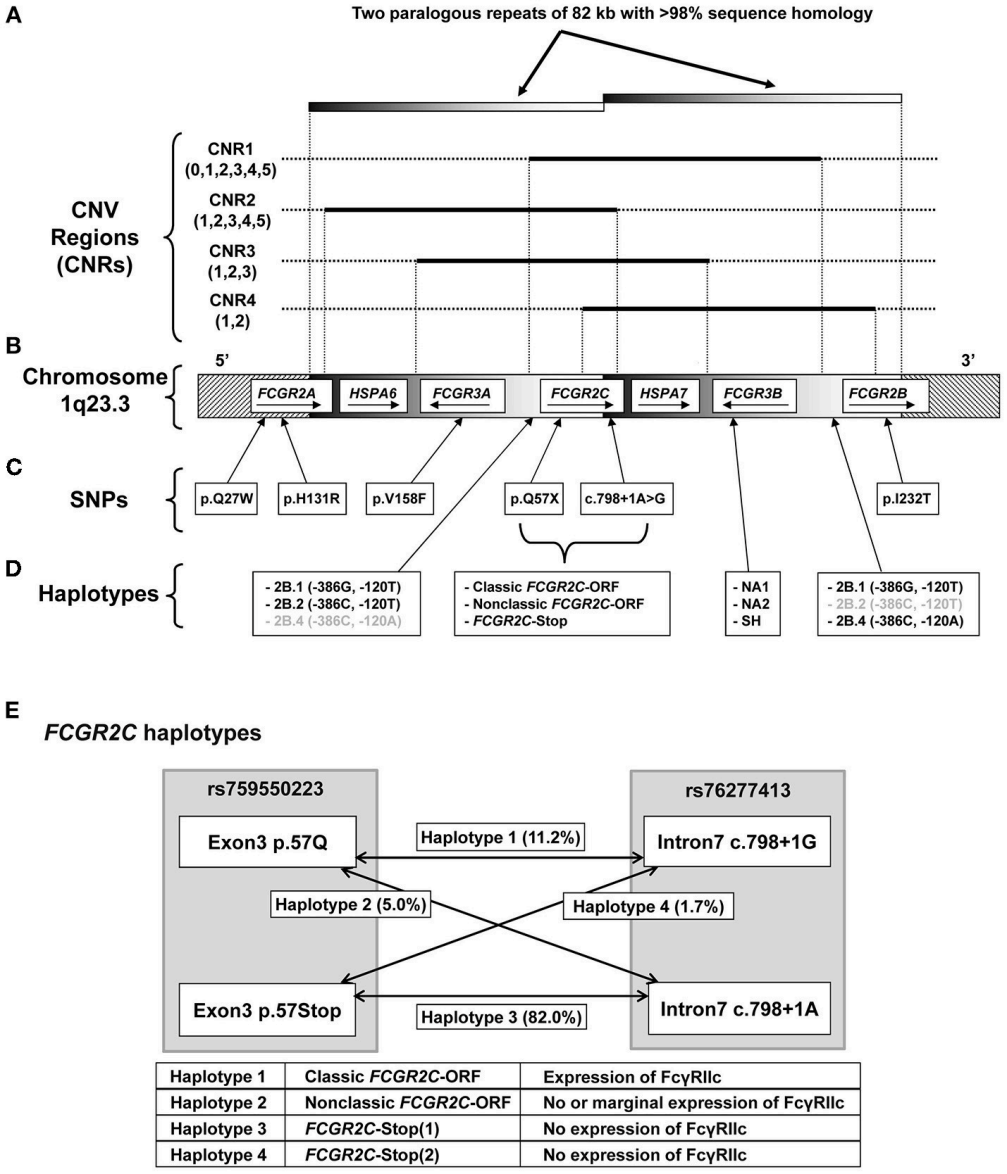
Genomic organization of the *FCGR2/3* locus. Overview of the *FCGR2/3* locus at 1q23.3. **(A)** CNV at the locus occurs always in regions containing a series of genes, termed CNV regions (CNRs) (18, 21). Numbers between brackets indicate observed copy number for that CNR in the individuals tested in this study, black lines indicate the extent of the different CNRs. Gray shaded bars indicate the extent of two paralogous repeats of the locus. The novel rare CNR4 that we recently described (18) was found in 4 of the individuals included in this study, and was combined with the similar CNR1 in this study for reasons of simplicity. **(B)** Overview of the genes in the locus with their orientation. Depiction of genes is not to scale. **(C)** Functional SNPs at the locus, indicated by single letter amino acid code and amino acid position, except for the splice variant c.798+1A>G. All these SNPs were determined in this study. **(D)** Functional haplotypes at the locus. 2B.1, 2B.2, and 2B.4 are haplotypes of two SNPs (at nucleotide positions −386 and −120 relative to the start of translation) in the otherwise identical promoter regions of *FCGR2B* and *FCGR2C* (haplotypes in gray are very rarely found in that particular gene). *FCGR3B* NA1, NA2, and SH are haplotypes determined by six SNPs. These haplotypes determine the different allotypes of Human Neutrophil Antigen 1 (HNA1) involved in neutrophil alloimmunization, and respectively encode the HNA1a/HNA1b/HNA1c antigenic variants. **(E)** Schematic representation of the different haplotypes of *FCGR2C* haplotypes, which determine expression of FcγRIIc. *FCGR2C*-Stop (1) and *FCGR2C*-Stop (2) haplotypes are similar in function and expression and are taken together as *FCGR2C*-Stop throughout the manuscript. Percentages represent allele frequencies of the different haplotypes in European healthy controls.

The *FCGR2/3* locus is a complex region due to the presence of a large segmental duplication and copy number variants (CNV) (18, 43). MLPA was previously shown to accurately call copy number variation at the *FCGR2/3* locus (5, 20). We used the MLPA to accurately identify all eight known functional SNPs and haplotypes, as well as the four CNV regions (CNRs), at the *FCGR2/3* locus, which have previously been associated with various autoimmune and infectious diseases (Figure 1 and Table S2).

### Allele Frequencies of CNV and SNPs at the *FCGR2/3* Locus Vary Among Different Ethnic Groups, Especially for the Classic and Nonclassic *FCGR2C*-ORF Haplotypes

The frequencies of many of the functional SNPs and CNVs have been reported to vary among different ethnic backgrounds (10, 21, 44–47), but information about the *FCGR2C* haplotypes is yet to be established. To explore differences in frequencies of SNPs and CNRs between several ethnic groups, we genotyped and compared large groups of healthy human subjects. Significant differences (*P* < 0.05) between ethnic groups were found for CNRs and for all SNPs except the *FCGR3A*-V158F SNP, which had no difference in frequency among all groups (Table 1). Analysis of subgroups within the European and African populations revealed subtle differences within the European population and marked differences within the African population (Table S3).

**Table 1:** Frequencies of CNVs (CNRs, proportion of individuals with that number of copies is shown) and SNPs (allele frequencies are shown).

**Variant**		**European (*n* = 919)**	**Chinese (*n* = 428)**	**African (*n* = 508)**	**Fisher's exact**
**CNR1**					
*FCGR3B + FCGR2C*	0 copies	0.00	0.00	0.00	
	1 copy	0.07	0.09	0.11	
	2 copies	0.83	0.73	0.73	
	3 copies	0.09	0.17	0.14	
	4 copies	0.01	0.01	0.01	**<0.0001**
**CNR2**					
*FCGR3A + FCGR2C*	1 copy	0.01	0.01	0.01	
	2 copies	0.94	0.96	0.96	
	3 copies	0.04	0.04	0.03	
	4 copies	0.00	0.00	0.00	0.87
**CNR3**					
*FCGR3A + FCGR2C*	1 copy	0.00^*^	0.00	0.00^*^	
	2 copies	1.00	0.98	1.00	
	3 copies	0.00	0.02	0.00	**<0.001**
***FCGR2A***					
	131 H	0.54	0.67	0.44	
	131 R	0.46	0.33	0.56	**<0.0001**
	27 Q	0.88	1.00	0.89	
	27 W	0.12	0.00	0.11	**<0.0001**
***FCGR3A***					
	158 F	0.64	0.64	0.64	
	158 V	0.36	0.36	0.36	0.94
***FCGR2C***					
	Stop	0.84	1.00	0.90	
	Classic ORF	0.11	0.00	0.02	
	Nonclassic ORF	0.05	0.00	0.08	**<0.0001**
Promoter haplotype	2B.1	0.89	1.00	0.95	
	2B.2	0.11	0.00	0.05	**<0.0001**
***FCGR3B***					
	NA1	0.35	0.62	0.38	
	NA2	0.62	0.38	0.46	
	SH	0.02	0.00	0.15	**<0.0001**
***FCGR2B***					
	232I	0.88	0.74	0.73	
	232T	0.12	0.26	0.27	**<0.0001**
Promoter haplotype	2B.1	0.90	1.00	0.99	
	2B.4	0.10	0.00	0.01	**<0.0001**

*Fisher's exact test: Overall P for differences between populations for that variation is shown. P-values < 0.05 are shown in bold*.

* *1 European and 1 West African individual showed a deletion of CNR3*.

**Figure 2:**
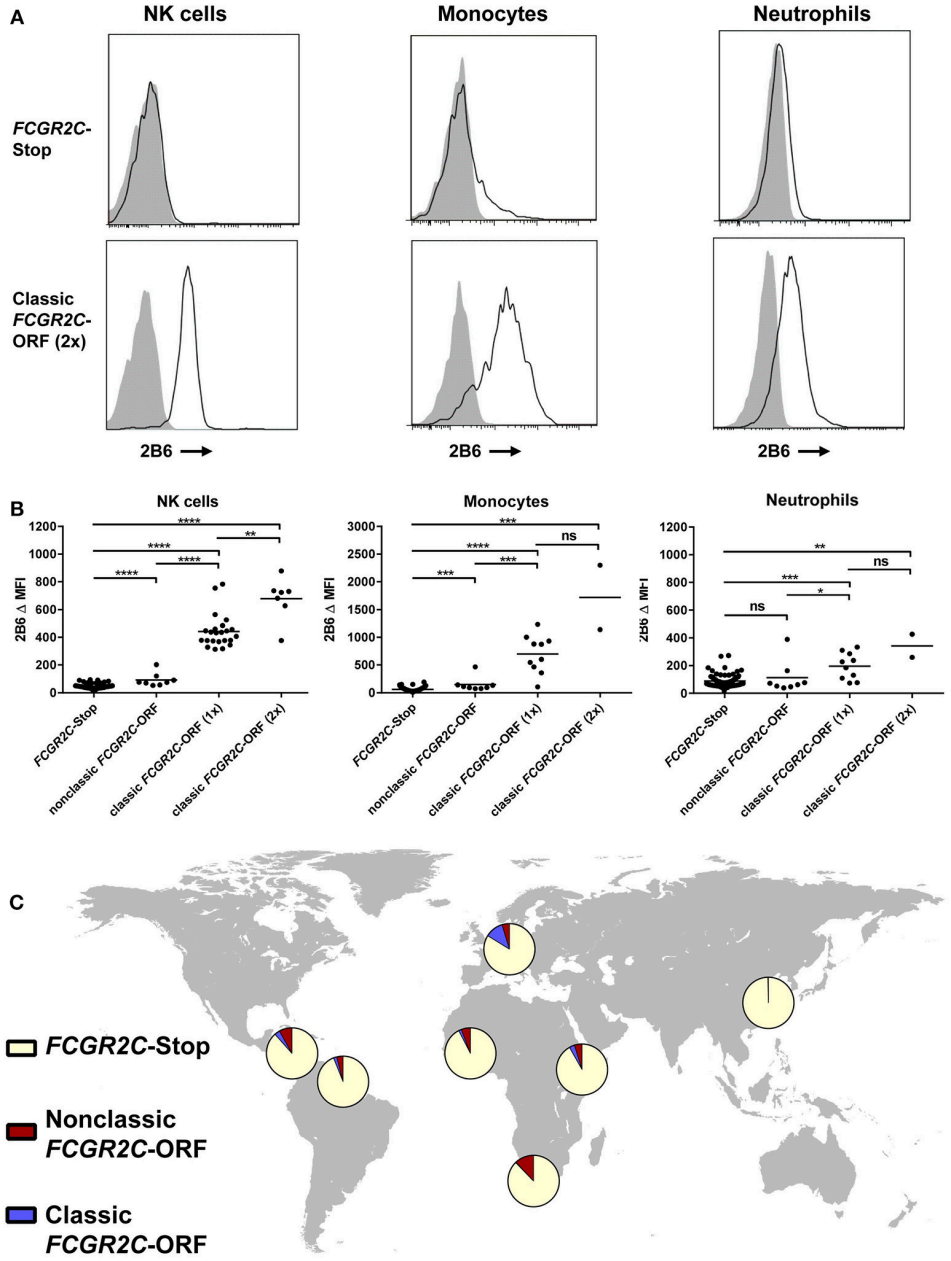
Haplotypes of *FCGR2C* determine expression of FcγRIIc. **(A)** Representative histograms of staining with MoAb 2B6 on NK cells (left), monocytes (middle), and neutrophils (right) of an individual homozygous for *FCGR2C*-Stop variant (upper panel) and an individual homozygous for the *FCGR2C*-ORF variant (lower panel). 2B6 recognizes the extracellular domain of both FcγRIIb and FcγRIIc. In *FCGR2C*-Stop individuals, FcγRIIc cannot be expressed, therefore staining of 2B6 in those individuals must be FcγRIIb only. Black line: 2B6, gray shading: isotype control. **(B)** Summary of 2B6 staining, corrected for isotype control, on human NK cells (left), monocytes (middle) and neutrophils (right) of genotyped individuals. Y axis scale is different for monocytes than for the other cell types. Because FcγRIIb is also stained by 2B6, only cells that do not express FcγRIIb can be easily analyzed for FcγRIIc expression. Therefore, individuals with a deletion of CNR1 were excluded from the analysis of NK cells, and individuals with a 2B.4 promoter haplotype in *FCGR2B* were excluded from the analysis of monocytes and neutrophils, because these variants result in ectopic expression of FcγRIIb on NK cells (35), or myeloid cells (36), respectively. NK cell analysis: *FCGR2C*-Stop *n* = 93, nonclassic *FCGR2C*-ORF, including cases with 1 or 2 copies *n* = 8, *FCGR2C*-ORF(1x), with 1 copy of the classic *FCGR2C*-ORF haplotype, *n* = 23, *FCGR2C*-ORF(2x), with 2 copies *n* = 7. Monocyte and neutrophil analysis: *FCGR2C*-Stop *n* = 99, nonclassic *FCGR2C*-ORF, including cases with 1 or 2 copies *n* = 8, *FCGR2C*-ORF(1x), with 1 copy of the classic *FCGR2C*-ORF haplotype, *n* = 10, *FCGR2C*-ORF(2x), with 2 copies *n* = 2. Some individuals were analyzed more than once at different time points with similar results; means are shown for these. All individuals analyzed are of European descent except for five *FCGR2C*-Stop and two nonclassic *FCGR2C*-ORF individuals who were of African origin. **(C)** World map showing allele frequencies of *FCGR2C* haplotypes for different ethnic groups. MFI, median fluorescence intensity; ns, non-significant; ^*^p < 0.05; ^**^p < 0.01; ^***^p < 0.001; ^****^p < 0.0001 as determined by Mann Whitney test.

Among the groups included, the largest difference in allele frequency was revealed for the *FCGR2C*-haplotypes. *FCGR2C* consists of three haplotypes; the *FCGR2C*-Stop pseudogene that is not expressed as a result of the *FCGR2C*-Q57X SNP (rs759550223), its expressed counterpart, the so-called classic *FCGR2C*-ORF with an open reading frame at rs759550223, and the nonclassic *FCGR2C*-ORF, which has an open reading frame at rs759550223 but has an almost complete lack of expression as a result of a splice site mutation in intron7 (rs76277413) (35). Figure 1E gives a schematic overview of the haplotypes of *FCGR2C*. The classic *FCGR2C*-ORF haplotype results in the expression of FcγRIIc as an activating IgG receptor on myeloid cells and NK cells, as we have characterized previously (5, 48). We now formally demonstrate that the nonclassic *FCGR2C*-ORF haplotype can be determined by MLPA (see Supplemental Methods and Table S4 for a description), as expression of FcγRIIc is indeed low to absent in individuals genotyped as nonclassic *FCGR2C*-ORF by MLPA (Figure 2, gating strategy Figure S1). The slight difference in staining levels compared to individuals with the *FCGR2C*-stop variant shows that there is some residual expression of FcγRIIc protein, but this is less than 10% of the expression in classic *FCGR2C*-ORF individuals. These haplotypes were markedly different among different ethnic groups; the classic *FCGR2C*-ORF haplotype was virtually absent in Chinese (present in 2 out of 428 individuals, minor allele frequency <0.005%) and rare in the different African populations, whereas the nonclassic *FCGR2C*-ORF was more prevalent in African populations compared to Europeans (Table 1 and Figure 2C).

### Linkage Disequilibrium at the *FCGR2/3* Locus Defined

**Figure 3:**
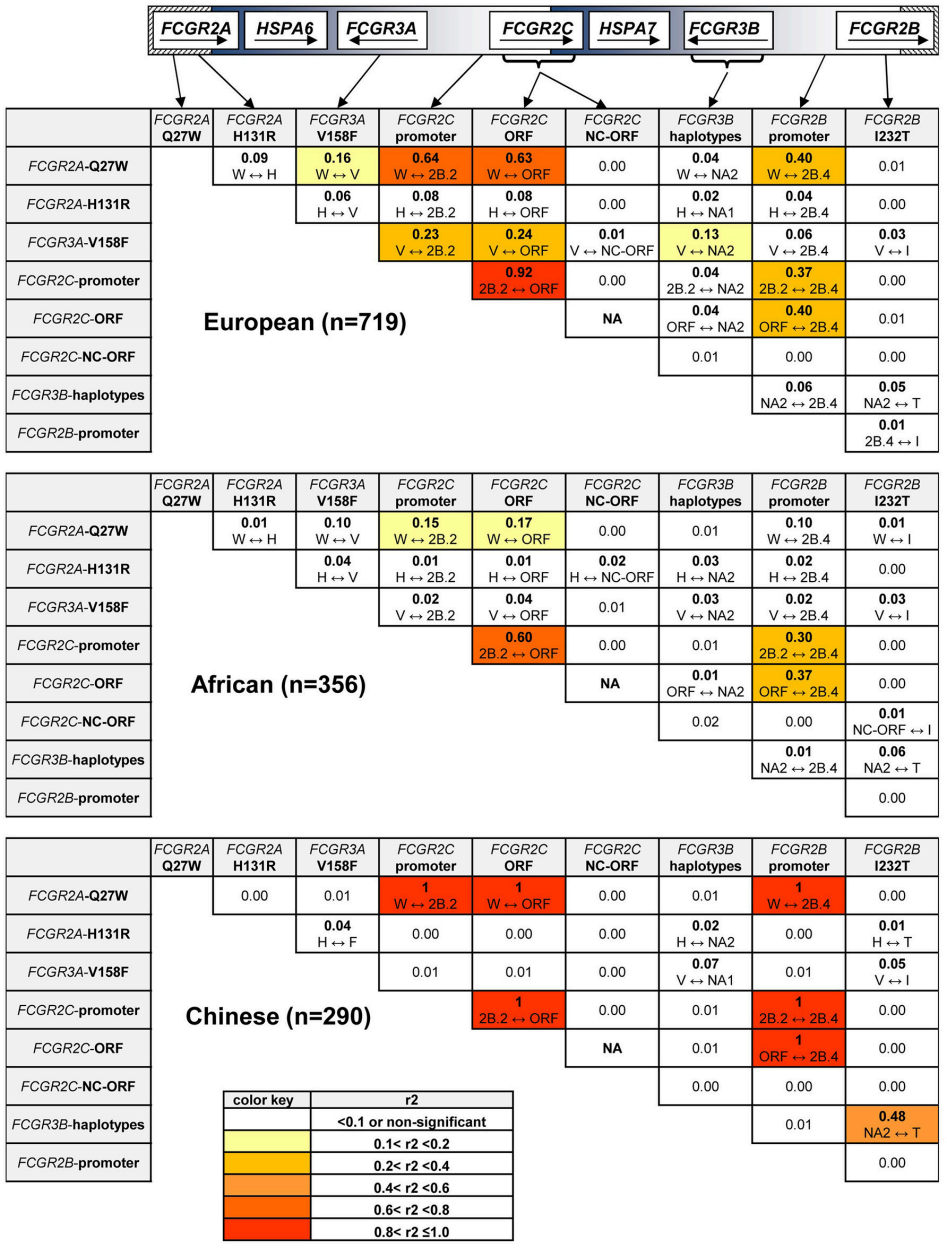
Linkage Disequilibrium at the *FCGR2/3* locus. Linkage Disequilibrium for SNPs and haplotypes in individuals without CNV. *r*^2^ is shown for all combinations, which variant is linked to which variant is shown underneath. Values shown in bold are significantly different from 0 (*p* < 0.05). *FCGR2C*-ORF = classic *FCGR2C*-ORF haplotype vs. all other *FCGR2C* haplotypes. *FCGR2C*-NC-ORF = nonclassic *FCGR2C*-ORF haplotype vs. all other *FCGR2C* haplotypes. NA, not available, because the classic *FCGR2C*-ORF haplotype and nonclassic *FCGR2C*-ORF haplotype are mutually exclusive. Polymorphic amino acids are indicated by one-letter code.

Because many functionally relevant SNPs in the *FCGR2/3* locus are located in close proximity to each other, the SNPs in *FCGR* genes are likely to be in strong LD, which can greatly complicate the interpretation of genetic association studies. From the control samples of the different ethnic reference populations, we first calculated the background LD pattern based on the SNPs and haplotypes in the individuals that did not show CNV (*r*^2^ in Figure 3, D' in Figure S2).

In the European population, we found strong LD of the classic *FCGR2C*-ORF haplotype (rs759550223 and rs76277413) with several of the other SNPs in the region. First, the classic *FCGR2C*-ORF haplotype was in almost complete LD (*r*^2^ = 0.92) with the 2B.2 promoter in *FCGR2C* (rs149754834). Furthermore, there was strong LD between the classic *FCGR2C*-ORF variant and *FCGR2A*-27W (rs201218628, *r*^2^ = 0.63) and with the 2B.4 promoter haplotype in *FCGR2B* (rs143796418, *r*^2^ = 0.40). Weaker LD was observed for the classic *FCGR2C*-ORF haplotype with *FCGR3A*-158V (rs396991, *r*^2^ = 0.24) and *FCGR2A-*131H (rs1801274, *r*^2^ = 0.08).

In the Chinese population, LD for the classic *FCGR2C*-ORF haplotype appeared similar to the LD in Europeans, but this was based only on 2 individuals.

In the African population, LD was also found for the *FCGR2C*-ORF haplotype with several of the variants, but in general this LD was weaker than in Europeans (Figure 3, second panel).

The previously described LD between *FCGR3A*-158V (rs396991) and *FCGR2A-*131H (rs1801274) (21, 23) was confirmed in the European and African population, although relatively weak (*r*^2^ = 0.06). We show now that this LD was reversed in the Chinese population, i.e., *FCGR3A*-158F (rs396991) and *FCGR2A*-131H (rs1801274) were in weak LD (*r*^2^ = 0.04).

We then investigated LD between CNV and SNPs for all of the CNRs known at the locus. Because the standard measurements of LD (*r*^2^ and D') cannot be calculated in areas with CNV, we performed this analysis by calculating allele frequencies for groups of individuals with normal (2 copies), decreased (≤1 copies) or increased (≥3 copies) copy number of at least one CNR and analyzed significant differences by Fisher's exact test.

Results for CNR1 are shown in Table S5. For CNR1, strong LD was found between increased copy number and the nonclassic *FCGR2C*-ORF haplotype (rs759550223 and rs76277413), both in the European and African population. Increased copy number in CNR1 also revealed strong LD with the *FCGR3B*-SH (rs5030738) haplotype in the European, but not in the African population. Some other SNPs [*FCGR2A*-H131R (rs1801274); *FCGR3A*-V158F (rs396991); *FCGR2B*-I232T (rs1050501)] were also associated with changes in CNV in CNR1.

For the less prevalent CNR2, LD was found only for rs1050501 in the European population (All results for CNR2 are shown in Table S6).

For the rare CNR3, no statistically significant LD was found at all (data not shown).

### Association of SNPs and CNV at the *FCGR2/3* Locus With Susceptibility to KD

After defining the background allele frequencies and LD of the functional SNPs and CNV in the control groups, we then analyzed the full content of variants in the *FCGR2/3* locus for susceptibility to KD, now also including the SNPs and CNV in the region that had not been covered in our previous GWAS study (6). We performed a case-control study in 405 KD cases and the cohort of 919 controls described above, all of European descent. For a family-based association study, 586 complete trios and 37 incomplete trios were genotyped. The characteristics of the KD patients are shown in Table S7.

### Case-Control Study

Genotype and allele frequencies of CNVs and SNPs are shown in Table 2. Several significant differences between cases and controls were observed, the most significant being the classic *FCGR2C*-ORF (rs759550223 and rs76277413) (15.7% vs. 11.2%, *P* = 0.002). Other significantly associated SNPs were the 2B.2 promoter in *FCGR2C* (rs149754834) (15.3% vs. 10.8%, *P* = 0.009), the *FCGR2A* 27Q>W SNP (rs201218628) (15.3% vs. 11.9%, *P* = 0.014) and the 2B.4 promoter in *FCGR2B* (rs143796418) (12.7% vs. 10.0%, *P* = 0.047). These four significantly associated variants are in strong LD with each other (Figure 3). In a multiple logistic regression analysis that included all the variants, none were independently associated, but a backward regression analysis revealed the classic *FCGR2C*-ORF as the strongest predictor of KD susceptibility (data not shown).

**Table 2:** Genotype and allele frequencies of functional genetic variants at the *FCGR2/3* locus, comparing KD patients of European descent with healthy controls of European descent.

**Variant**	**Cases**	**Controls**	**Fisher**	**Single logistic regression (additive model)**		**Multiple logistic regression**	
	**(*n* = 405)**	**(*n* = 919)**				**All variants**	**2 variants**
			***P*-value**	**OR (95%LL-95%UL)**	***P*-value**	***P*-value**	***P*-value**
**CNR1**							
**(*****FCGR2C + FCGR3B*****)**							
0 copies	1	1		<2 vs. rest:			
1 copy	27	60		1.04 (0.66–1.66)	0.853	0.719	
2 copies	348	768					
3 copies	27	83		>2 vs. rest:			
4 copies	2	7	0.533	0.71 (0.46–1.10)	0.124	0.291	
**CNR2**							
**(*****FCGR2C + FCGR3A*****)**							
1 copy	3	11		<2 vs. rest:			
2 copies	376	866		0.62 (0.17–2.22)	0.459	0.491	
3 copies	25	41		>2 vs. rest:			
4 copies	1	1	0.390	1.43 (0.87–2.37)	0.162	0.256	
**CNR3**							
**(*****FCGR2C + FCGR3A*****)**							
2 copies	405	917		>2 vs. rest:			
3 copies	0	2	1.000	0.00 (0.00-inf)	0.973	0.973	
***FCGR2A*** **Q27W**							
QQ	289	713					
QW	108	194					
WW	8	12	**0.047**				
Allele frequency (W)	15.3%	11.9%		**1.35 (1.06–1.72)**	**0.014**	0.783	
***FCGR2A*** **H131R**							
HH	122	269					
HR	211	463					
RR	72	187	0.559				
Allele frequency (H)	56.2%	54.5%		1.07 (0.91–1.27)	0.408	0.857	0.927
***FCGR3A*** **V158F**							
0 V (F, FF, FFF, FFFF)	150	386					
1 V (V, VF, VFF)	205	403					
2 V (VV, VVF, VVFF)	47	128					
3 V (VVV)	3	2	**0.046**				
Allele frequency (V)	37.0%	35.5%		1.08 (0.91–1.28)	0.373	0.606	
***FCGR2C*** **promoter**							
0 2B.2	286	717					
1 2B.2	110	185					
2 2B.2	9	16					
3 2B.2	0	1	**0.017**				
Allele frequency (2B.2)	15.3%	11.5%		**1.37 (1.08–1.72)**	**0.009**	NE	
***FCGR2C***							
**ORF/Stop/NC-ORF**							
0 ORF	283	721					
1 ORF	113	184					
2 ORF	9	13					
3 ORF	0	1	**0.005**				
0 NC-ORF	389	853					
1 NC-ORF	6	33					
2 NC-ORF	10	33	0.059				
Allele frequency (ORF)	15.7%	11.2%		**1.46 (1.16–1.85)**	**0.002**	0.093	**0.002**
Allele frequency (NC-ORF)	3.1%	5.2%		0.72 (0.51–1.02)	0.063	0.112	
Allele frequency (Stop)	81.2%	83.7%		0.88 (0.74–1.04)	0.136		
***FCGR3B*** **NA1/NA2/SH**							
0 NA1	158	373					
1 NA1	201	430					
2 NA1	45	114					
3 NA1	1	2	0.754				
0 SH	389	874					
1 SH	16	45	0.481				
Allele frequency (NA1)	36.2%	35.3%		1.01 (0.85–1.20)	0.933	0.537	
Allele frequency (NA2)	63.8%	64.7%		0.94 (0.80–1.12)			
Allele frequency (SH)	4.0%	4.9%		0.80 (0.45–1.43)	0.450	0.247	
***FCGR2B*** **promoter**							
0 2B.4	307	748					
1 2B.4	93	157					
2 2B.4	5	14	**0.043**				
Allele frequency (2B.4)	12.7%	10.0%		**1.29 (1.00–1.67)**	**0.047**	0.834	
***FCGR2B*** **I232T**							
II	322	697					
IT	76	201					
TT	7	21	0.359				
Allele frequency (T)	11.1%	13.2%		0.83 (0.64–1.06)	0.141	0.189	

*For SNPs that are subject to CNV, several genotypes are pooled as indicated to combine all the different genotypes with the same copy number of 1 of the variants. For the tri-allelic haplotypes in FCGR2C and FCGR3B, this is done for two of the haplotypes separately. Fisher exact test was calculated on genotype frequencies as shown in the table. A single logistic regression analysis was performed for each (presumed) risk allele in an additive model. A multiple logistic regression analysis was performed on all variants (except the FCGR2C promoter haplotypes, which were left out of the multiple logistic regression analysis because of the near perfect LD with the classic FCGR2C-ORF haplotype) and on FCGR2A-H131R and classic FCGR2C-ORF alone. P-values < 0.05 are shown in bold*.

We did not detect significant differences for any of the CNV regions, or for the other functional SNPs. Even though we detected a slight trend among the KD patients with higher frequency of the *FCGR2A*-131H (rs1801274) risk allele in the current study, this association found previously in GWAS and meta-analysis (6, 7, 33) was not replicated in this dataset of European patients and healthy controls. A multiple logistic regression analysis of only the *FCGR2C*-ORF and *FCGR2A*-131H revealed that the association of *FCGR2C*-ORF was independent of *FCGR2A*-131H (Table 2).

### Family-Based Study on KD

In an attempt to confirm our findings, we performed a KD family-based association study in 623 family trios in which the child was diagnosed with KD. The transmission disequilibrium test (TDT) analysis revealed a significant association (*P* = 0.006) of *FCGR2A*-131H (rs1801274) (Table 3). For the *FCGR2C*-ORF haplotype (rs759550223 and rs76277413) and the other SNPs or CNRs tested, there was no evidence of association (except for the rare allele with two copies of *FCCR3A* on one chromosome, of which one was 158V and the other was 158F, which had only 18 informative families) (Table 3). Of note, the number of informative families for *FCGR2C*-ORF was also relatively small, as a result of the low prevalence of this variant (Table 1). Analysis of the families enabled us to construct complete haplotypes for all parental chromosomes, which confirmed the LD pattern observed in the cohort of healthy controls, both in parents without any CNV as in parents that did show CNV (Figure S3).

**Table 3:** Transmission disequilibrium test for the different variants at the *FCGR2/3* locus in a family-based association study.

**Allele/haplotype (on 1 chromosome)**	**Allele frequency**	# families^*^	***Z***	***P*-value**
**CNR1**				
0 (deletion)	0.049	105	0.285	0.776
1	0.875	214	−0.065	0.948
2 (duplication)	0.074	133	−0.338	0.735
**CNR2**				
0 (deletion)	0.006	13	−0.277	0.782
1	0.976	61	0.378	0.705
2 (duplication)	0.018	48	−0.429	0.668
***FCGR2A*** **Q27W**				
Q	0.891	210	−0.328	0.743
W	0.109	210	0.328	0.743
***FCGR2A*** **H131R**				
H	0.575	431	2.750	**0.006**
R	0.425	431	−2.750	**0.006**
***FCGR3A*** **V158F**				
–	0.005	11	−0.302	0.763
F	0.642	395	0.483	0.629
FF	0.010	27	0.577	0.564
VF	0.006	18	−2.828	**0.005**
V	0.331	397	−0.088	0.930
VV	0.004	10	0	1.000
**Promoter** ***FCGR2C***				
-	0.055	114	0.451	0.652
2B.1	0.748	349	−0.242	0.809
2B.1-2B.1	0.089	164	−0.818	0.414
2B.2	0.099	182	0.491	0.623
***FCGR2C*** ORF/Stop/NC-ORF^**^				
–	0.055	115	0.268	0.788
ORF	0.100	184	1.120	0.263
Stop	0.743	354	−0.241	0.810
NC-ORF	0.009	23	−1.460	0.144
Stop-stop	0.075	143	−0.477	0.633
NC-ORF-NC-ORF	0.007	19	−0.229	0.819
***FCGR3B*** **NA1/NA2/SH**				
–	0.053	108	0.186	0.853
NA1	0.362	389	0.490	0.624
NA1-NA2	0.051	96	−0.198	0.843
NA1-SH	0.009	21	0.218	0.827
NA2	0.508	396	−0.439	0.660
NA2-NA2	0.004	11	−0.905	0.366
SH	0.004	12	−1.155	0.248
**Promoter** ***FCGR2B***				
2B.1	0.905	185	−0.563	0.574
2B.4	0.090	172	0.946	0.344
***FCGR2B*** **I232T**				
I	0.867	226	0.741	0.459
T	0.133	226	−0.741	0.459

* *Number of informative families (i.e., at least one of the parents is heterozygous for the indicated allele or haplotype). Only alleles for which the number of informative families is >10 are shown*.

** *ORF means classic FCGR2C-ORF haplotype, NC-ORF means nonclassic FCGR2C-ORF haplotype. Z; Z statistic, a positive Z indicates more transmission than expected, a negative Z indicates less transmission than expected, P indicates whether Z is significantly different from 0, P < 0.05 is considered significant*.

### Combined Analysis Reveals Both *FCGR2A*-131H and *FCGR2C*-ORF to be Significantly Associated With Susceptibility to KD

**Figure 4:**
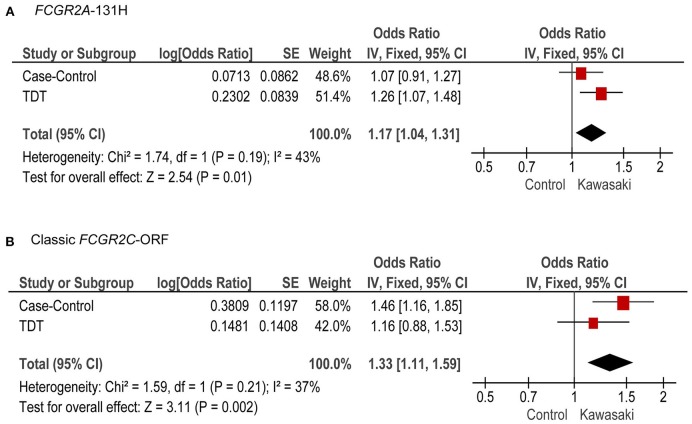
Meta-analysis of case-control and TDT for *FCGR2A*-131H and the classic *FCGR2C*-ORF haplotype. Combined OR (95% CI) and *P*-values from the case-control study and TDT analysis, for *FCGR2A*-131H **(A)** and classic *FCGR2C*-ORF **(B)**.

We performed a meta-analysis of the associations from both the case-control and familial TDT analyses, and we found the classic *FCGR2C*-ORF haplotype (rs759550223 and rs76277413, meta-P = 0.002) and the *FCGR2A* 131H (rs1801274, meta-P = 0.01) were both significantly associated with KD susceptibility (Figure 4).

### mRNA for the FCGR2 Isoforms Is Upregulated in Acute KD Patients, in Contrast to the FCGR3 Isoforms

**Figure 5:**
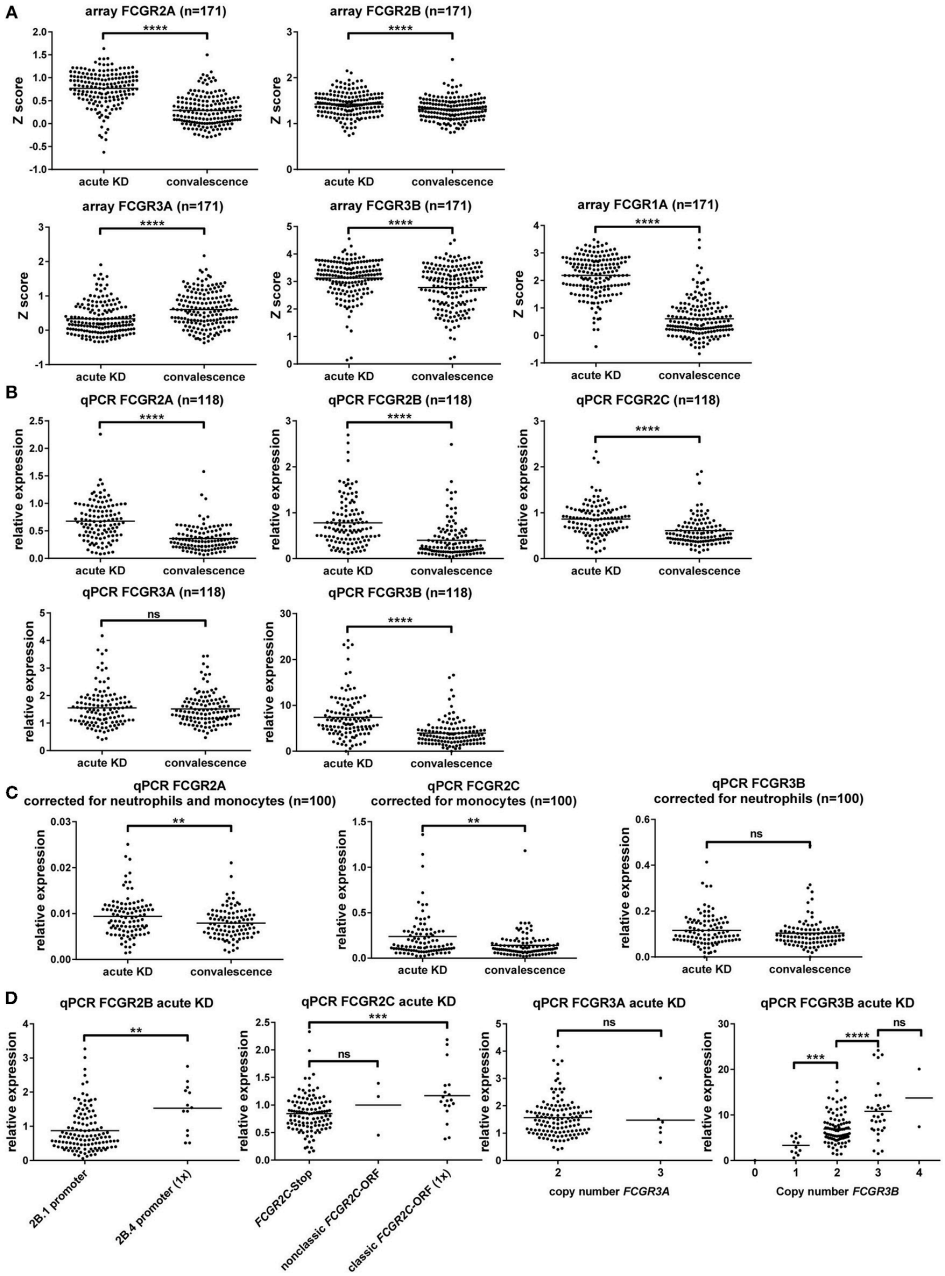
Gene expression analysis of FcγRs shows upregulation of FcγRI and FcγRII, but not FcγRIII during acute KD. **(A)** Difference in expression intensity of various FCGR transcripts, as determined by RNA microarray, shown as Z scores (higher score indicating higher expression), in 171 subjects with KD in the acute and convalescent phase of the disease. **(B–D)** Relative expression of different FCGR transcripts detected by qPCR on whole blood, corrected for housekeeping genes GUS and GAPDH, as compared to one randomly chosen sample in the convalescence phase of KD. **(B)** Dot plots showing a comparison of the acute and convalescent phase of KD in 118 patients. **(C)** Dot plots showing a comparison of the acute and convalescent phase of KD for transcripts of FCGR2A, FCGR2C, and FCGR3B in 100 patients for which WBC differentials were known, after correction for the main cell type that expresses the transcript. **(D)** Comparison of genotypes for the expression of various transcripts in 135 patients with acute KD. FCGR2B: patients with only the 2B.1 promoter (*n* = 122) or with 1 copy of the 2B.4 promoter (*n* = 13) in *FCGR2B*. FCGR2C: patients with the *FCGR2C*-Stop haplotype (*n* = 114), patients with 1 copy of the classic *FCGR2C*-ORF haplotype (*n* = 18), patients with 1 or 2 copies of the nonclassic *FCGR2C*-ORF haplotype (*n* = 3). FCGR3A patients with 2 copies (*n* = 129), or 3 copies (*n* = 6) of the *FCGR3A* gene. FCGR3B: patients with 0 copies (*n* = 1), 1 copy (*n* = 12), 2 copies (*n* = 89), 3 copies (*n* = 31) or 4 copies (*n* = 2) of the *FCGR3B* gene. ns: non-significant; ^**^p < 0.01; ^***^p < 0.001; ^****^p < 0.0001 as determined by paired *t*-test or Wilcoxon matched-pairs signed rank test **(A–C)** or students *t*-test or Mann Whitney test **(D)**.

To determine whether alteration of expression levels of the low-affinity FcγRs plays a role in the pathophysiology of KD, we compared mRNA expression levels in KD patients in the acute and convalescent phase of the disease, using samples from a previous study (49). First, we compared Z scores for FCGR transcripts that were already present in the microarray for this study. In this analysis, we found FCGR2A, FCGR2B, FCGR3A, FCGR3B, and also FCGR1A, encoding the high-affinity FcγRI, to be all transcriptionally upregulated in acute KD (Figure 5A).

To confirm these findings and extend the analysis to FCGR2C, we then performed highly specific qPCRs for FCGRs on a selection of these patients from which RNA was still available. This confirmed that FCGR2A, FCGR2B and FCGR2C transcripts were all upregulated during acute KD (Figure 5B). FCGR3A was not differentially expressed between the acute and convalescent phase (Figure 5B) but FCGR3B seemed to be upregulated in the acute phase (Figure 5B). However, because acute KD could have resulted in a shift in leukocyte differentials and in our cohort a marked increase of neutrophil percentages was observed (data not shown), we applied a correction for percentages of different leukocyte subsets in the 100 patients for whom leukocyte differentials were available. In the case of FCGR3B, a correction for neutrophil percentages (Figure 5C) showed that the apparent upregulation was the result of the relative increase in neutrophils during acute KD and does not reflect a true increase in transcription. On the other hand, expression levels of FCGR2A and FCGR2C were increased in acute KD even after correction for shifts in white blood cell distribution (Figure 5C).

Comparison for several genetic differences known to influence expression levels showed marked differences (Figure 5D), confirming earlier reports and the validity of our analysis.

## Discussion

In a comprehensive study using MLPA, we have analyzed the full collection of functionally defined SNPs and CNRs at the *FCGR2/3* locus at an unprecedented level of detail. We report extensive LD in this notoriously difficult gene cluster, as well as large ethnic variation in different European, African and Asian subpopulations. Our findings are in line with previously published allele frequencies and CNV in different populations for this locus (21, 44, 50) and extend these findings with additional variants and populations. Applying this as the reference dataset, previously reported genetic association studies may need to be re-evaluated.

This is the first study to illustrate the relevance of a more detailed reference for a pediatric vasculitis. KD has a ten-fold increased prevalence in Japanese and other Asian populations compared to children of European descent. In multi-ethnic GWAS studies, the association of *FCGR2A*-131H(rs1801274) with KD susceptibility was detected across KD cohorts of different ethnic backgrounds, indicating that this common variant is an independent susceptibility marker in all groups, including the Asian and European populations (6, 7). We now show that within the European cohorts, the classic *FCGR2C*-ORF haplotype (rs759550223 and rs76277413) may be the most strongly associated *FCGR* gene variant with KD susceptibility. Evidence from low LD (*r*^2^ = 0.08) and conditional analyses identify the association of this classic *FCGR2C*-ORF haplotype to be independent of the previously identified *FCGR2A*-131H GWAS association. Interestingly, the classic *FCGR2C*-ORF, which is strongly associated with KD susceptibility in Europeans, was virtually non-existent in the Asian populations. This suggests that the increased prevalence of KD in Asian populations compared to European populations derives from factors other than the currently known genetic variation in *FCGR* genes.

The very strong LD of the classic *FCGR2C*-ORF haplotype with several other variants in the *FCGR2/3* locus means that the interpretation of associations with this locus are more complex than previously appreciated. Classic *FCGR2C*-ORF is in strong LD with three other variants: the 2B.2 promoter in *FCGR2C* (rs149754834), *FCGR2A*-27W (rs201218628) and the 2B.4 haplotype in *FCGR2B* (rs143796418). Hence, all these variants could tag the classic *FCGR2C*-ORF and were also significantly associated with KD susceptibility in a single logistic regression analysis. However, when we analyzed all variants in a multiple logistic regression analysis, we found the classic *FCGR2C*-ORF to be the strongest predictor of KD susceptibility. The 2B.2 variant in *FCGR2C* was omitted from the multiple logistic regression analysis because of its near complete LD with classic *FCGR2C*-ORF. In fact, this variant can actually be only of biological relevance in the case of a classic *FCGR2C*-ORF haplotype, because with the other *FCGR2C* haplotypes, this 2B.2 promoter haplotype would reside in the promoter of an untranslated variant or *FCGR2C* (*FCGR2C*-Stop or nonclassic *FCGR2C*-ORF). It is unlikely that the tagging *FCGR2A*-Q27W SNP independently contributes to KD susceptibility, as it is a genetic variation for which a biological role has not been described (46). It lies outside the IgG-binding region of FcγRIIa and an analysis of expression levels revealed no influence on expression levels (Figure S4). However, genotyping the *FCGR2A*-Q27W SNP may be informative in genetic association studies, as it may be used as a tagging SNP for the classic *FCGR2C*-ORF as part of a susceptibility haplotype. The *FCGR2A*-Q27W SNP lies outside the copy number variable part of the *FCGR2/3* locus and is straightforward to genotype.

We did not find a significant association of CNV of the locus for any of the different CNRs that have been described. This is in contrast with an earlier report that described an association of CNV in *FCGR3B* and in *FCGR2C* with susceptibility to KD (51). In our opinion, analysis of CNV of *FCGR2C* without information on the *FCGR2C*-ORF variant is futile, as CNV of *FCGR2C per se* does not correlate with expression levels, normally being a pseudogene (i.e., *FCGR2C*-Stop). On the other hand, CNV in the *FCGR3B* does have a potential biological role, as we confirmed with our qPCR analysis, which showed a direct effect of CNV of the *FCGR3B* gene on transcript levels of FCGR3B. Nevertheless, CNV of *FCGR3B* was not associated with KD susceptibility in our cohorts.

Transcript levels of FCGR2A have previously been shown to be increased in KD patients compared to febrile controls (52), and we now show that mRNA levels of all FCGR2 isoforms, as well as FCGR1A1 [encoding FcγRI (CD64)], are upregulated during the acute phase of KD, compared to paired convalescent samples of the same patients, which further underscores the importance of FcγRs in KD.

A striking finding of our study is the lack of a significant association of *FCGR2A*-131H in the case-control study, contrasting our previous GWAS findings (6). This discrepancy was not explained by a difference in allele frequency in the case group, but by a difference in allele frequency between the control groups tested. Both control groups were randomly selected individuals of European descent. A remarkable difference between the two control groups was that the control group of the GWAS consisted mainly of individuals from the United Kingdom, which in the present study have a significantly lower prevalence of the *FCGR2A*-131H than the other European groups (Table S3). Apparently, even within the European population, the selection of the control group may influence the results of association analyses. Although both control groups were randomly selected, we believe that the group used in the current study is more representative of the background population, since it consists of more controls from the countries of origin of the patients. Nevertheless, even with the new control group, in a combined meta-analysis with our TDT analysis, *FCGR2A*-131H was still significantly associated with KD susceptibility.

In addition to small differences within the European population, of more relevance were the significant differences in allele frequencies at the *FCGR2/3* locus between the different ethnic groups. Our MLPA assay enabled us to look at the distribution of *FCGR2C* haplotypes in African, European and Chinese populations. We show that MLPA reliably distinguished the classic *FCGR2C*-ORF from the nonclassic *FCGR2C*-ORF haplotype that does not result in expression of FcγRIIc. Theoretically, only minimal errors in haplotype calling can occur for *FCGR2C* with the MLPA methods (calculated error rate of only 0.1%, Table S4), whereas Illumina whole-exome sequencing was unable to detect the rs759550223 SNP of the classic *FCGR2C*-ORF haplotype in all three individuals with this haplotype among ten individuals tested in total (error rate 30%) (18).

The classic *FCGR2C*-ORF haplotype is virtually absent from the Asian population, whereas in the African population, the non-expressed nonclassic ORF was much more prevalent than the classic *FCGR2C*-ORF. The absence of the classic *FCGR2C*-ORF in the Asian population is of particular interest because of the fact that there is a striking difference in the incidence of KD between children of Asian (69–308 per 100,000 children <5 years of age) (53) and of European descent (4–15 per 100,000 children <5 years of age) (54–56). Clearly, the *FCGR2C*-ORF is only a risk factor for KD susceptibility in European subjects, and cannot account for the increased incidence of KD in Asian children.

A potential limitation of our MLPA technology lies in the uncertainty of allocating the promoter haplotypes 2B.2 and 2B.4 to either *FCGR2B* or *FCGR2C*, but data previously generated by us and others (5, 36, 57, 58) show that our allocation approach is accurate in >95% of European individuals with at least one of the rare variants 2B.2 or 2B.4. The majority of individuals does not carry a rare variant and these individuals will be 100% accurately genotyped by MLPA.

Detailed knowledge of genetic linkage in IgG receptors has major implications for every other study on associations of *FCGR2/3* polymorphisms with disease or therapeutic efficacy. For example, many studies investigating associations with therapeutic efficacy of therapeutic antibodies against cancer have found an association with the *FCGR3A*-158V variant (rs396991) (13–15, 59), which we now show to be in moderate LD with the classic *FCGR2C*-ORF (*r*^2^ = 0.24). Since the classic *FCGR2C*-ORF haplotype leads to expression of the activating FcγRIIc on NK cells, neutrophils, monocytes (Figure 2) and macrophages (17), it may contribute to killing of tumor cells by antibody-dependent cellular cytotoxicity by these cells, and could potentially be a stronger predictor of treatment success.

In conclusion, we have reported a novel association of the classic *FCGR2C*-ORF variant (rs759550223 and rs76277413) with susceptibility to KD in European patients, independent of the *FCGR2A*-131H (rs1801274), which is a separate susceptibility marker. Upregulation of the transcripts for both activating receptors encoded by these genes (respectively FcγRIIc and FcγRIIa) during acute KD further indicates their importance in KD pathophysiology. FcγRIIa and FcγRIIc are co-expressed by two circulating cell types, monocytes and neutrophils. Both cell types are actively recruited to arterial lesions in KD patients. Our data support a central role of the activating IgG receptors on these cell types in the pathophysiology of KD, whereas the SNPs in the inhibitory FcγRIIb were not associated. This suggests that inhibiting the function of activating FcγRs (which is a possible working mechanism of IVIg, the first-line treatment in KD) may be an important treatment goal in patients with this pediatric vasculitis during the acute phase of the disease.

## Author Contributions

SN and CT performed experiments, analyzed data, wrote the manuscript and designed research. WB discussed data and designed research. MT performed statistical analysis. JG, LH, EP, AN, and JvdH performed experiments and analyzed data. RY, ML, VW, DB, A-LP, JE, RC, CS, JB, KF, and CvdS provided samples. TvdB, SD, and MH supervised research. MdB discussed data and designed research. TK supervised the study, wrote the manuscript and designed research. All authors contributed to the final manuscript.

### Conflict of Interest Statement

The authors declare that the research was conducted in the absence of any commercial or financial relationships that could be construed as a potential conflict of interest.
