# Supplementary material for: Extensive Ethnic Variation and Linkage Disequilibrium at the FCGR2/3 Locus: Different Genetic Associations Revealed in Kawasaki Disease
Source: Front Immunol. 2019 Mar 21;10:185. doi: 10.3389/fimmu.2019.00185 (PMC6437109; doi:10.3389/fimmu.2019.00185)
Supplement: Supplementary file 1 [file Data_Sheet_1.doc]

Online supplemental tables, figures and methods for:

**Extensive ethnic variation and linkage disequilibrium at the *FCGR2/3* locus: different genetic associations revealed in Kawasaki Disease**

Sietse Q. Nagelkerke, Carline E. Tacke, Willemijn B. Breunis, Michael W.T. Tanck, Judy Geissler, Eileen Png, Long T. Hoang, Joris van der Heijden, Ahmad N.M. Naim, Rae S.M. Yeung, Michael L. Levin, Victoria J. Wright, David P. Burgner, Anne-Louise Ponsonby, Justine A. Ellis, Chisato Shimizu, Jane C. Burns, Karin Fijnvandraat, C. Ellen van der Schoot, Timo K. van den Berg, Martin de Boer, Sonia Davila, Martin L. Hibberd, Taco W. Kuijpers and the International Kawasaki Disease Genetics Consortium

**Table S1. Overview of subjects that were genotyped in the study**

|  | **Number** |
| --- | --- |
| **Case control study** | **1324** |
| **KD Cases** | 405 |
| Netherlands | 234 |
| Australia | 109 |
| USA | 62 |
| **Healthy controls** | 919 |
| European | 919 |
| Netherlands | 199 |
| Austria | 478 |
| Australia | 156 |
| United Kingdom | 86 |
| **Other healthy controls** | **936** |
| Chinese | 428 |
| African | 508 |
| South African | 149 |
| Ethiopian | 142 |
| West African | 65 |
| Surinam | 78 |
| Antillean | 74 |
| **Family-based association study** | **1831** |
| USA |  |
| KD patients | 386 |
| Parents | 734* |
| Australia |  |
| KD patients | 104 |
| Parents | 208 |
| Netherlands |  |
| KD patients | 98 |
| Parents | 196 |
| Italy |  |
| KD patients | 35 |
| Parents | 70 |
| **Total individuals** | **4091** |

* The cohort of USA subjects Family-based association study consisted of 348 complete trio’s and 38 incomplete trio’s in which DNA of only one parent was available for genotyping.

**Table S2. Overview of SNPs in the *FCGR2/3* locus included in the study.**

| **Rs #** | **Nucleotide*** | **amino acid**  **position**** | **amino acid** | **Functional change** |
| --- | --- | --- | --- | --- |
| ***FCGR2A*** |  |  |  |  |
| rs201218628 | c.184C c.185A | **27**(62) | Gln | no functional changes known |
|  | c.184T c.185G |  | Trp |  |
| rs1801274 | c.497A | **131**(166) | His | higher affinity for human IgG^1^ |
|  | c.497G |  | Arg |  |
| ***FCGR2B*** |  |  |  |  |
| rs143796418 | -386 C>G *** | - | - | Promoter haplotypes 2B.1, 2B.2 and 2B.4 influences expression^2-4^ |
| rs780467580 | -120 T>A *** | - | - |  |
| rs1050501 | c.695T | 188(**232**) | Ile |  |
|  | c.695C |  | Thr | Excludes receptor from lipid rafts^5, 6^ |
| ***FCGR2C*** |  |  |  |  |
| rs149754834 | -386 C>G *** | - | - | Promoter haplotypes 2B.1, 2B.2  functional change unknown |
| rs34701572 | -120 T>A *** | - | - |  |
| rs759550223 | c.169T | 13(**57**) | Ter |  |
|  | c.169C |  | Gln | Results in expression of FcγRIIc^7, 8^ |
| rs76277413 | c.798 +1 A>G | - | - | A causes exon7 to be spliced out^7^ |
| ***FCGR3A*** |  |  |  |  |
| rs396991 | c.526G | **158**(176) | Val | higher affinity for human IgG^1, 9^ |
|  | c.526T |  | Phe |  |
| ***FCGR3B*** |  |  |  |  |
| rs200688856 | c.108G | 18(**36**) | Arg | NA1**** |
|  | c.108C |  | Ser | NA2 and SH**** |
| rs527909462 | c.114C | 20(**38**) | Leu | NA1 |
|  | c.114T |  | Leu | NA2 and SH |
| rs448740 | c.194A | 47(**65**) | Asn | NA1 |
|  | c.194G |  | Ser | NA2 and SH |
| rs5030738 | c.233C | 60(**78**) | Ala | NA1 and NA2 |
|  | c.233A |  | Asp | SH |
| rs147574249 | c.244G | 64(**82**) | Asp | NA1 |
|  | c.244A |  | Asn | NA2 and SH |
| rs2290834 | c.316G | 88(**106**) | Val | NA1 |
|  | c.316A |  | Ile | NA2 and SH |

*

Nucleotide numbering excludes exon6 in FCGR2A and FCGR2C transcripts, because this exon is spliced out from these transcripts, but includes exon6 in FCGR2B, in which it is retained in many transcripts (splice variant known as FCGR2B1).

In FCGR2A transcripts, inconsistencies exist as a result of alternative splicing at the beginning of exon3 because of two adjacent splice acceptor sites that can both be used. The most commonly used amino-acid numbering is derived from the shorter transcript in which the 3’ splice acceptor site is used, so we chose to use this transcript for nucleotide numbering in this manuscript.

**

Inconsistencies exist in the amino-acid numbering used in the literature, because some SNPs are named by the position when including the signal peptides, and others are named by their position in the mature protein, excluding the signal peptides. In this table, position in the mature protein is shown first, followed by the position in the full protein between brackets. For the rest of the manuscript, we chose for each SNP to use the amino-acid position most often used, as indicated in bold in the table

***

relative to the start of translation

****

The set of 6 SNPs in *FCGR3B* determines the haplotypes for the human neutrophil antigen 1 (HNA1) that is involved in allo-immunization to neutrophils. Three major haplotypes exist: NA1, NA2 and SH, although additional variants exist^10^. The MLPA-probe used in the study to distinguish NA1 from NA2 has its ligation site at rs527909462.

**Table S3. Prevalence of CNVs and SNPs in the *FCGR2/3* locus within European and African subpopulations**

European

|  |  | **All European**  **(n=919)** |  | **Australia (n=156)** | **Austria**  **(n=478)** | **NL**  **(n=199)** | **UK**  **(n=86)** | **Fisher’s exact** |
| --- | --- | --- | --- | --- | --- | --- | --- | --- |
| **CNR1** |  |  |  |  |  |  |  |  |
| ***FCGR3B + FCGR2C*** | **0 copies** | 0.00 |  | 0.00 | 0.00 | 0.00 | 0.00 |  |
|  | **1 copy** | 0.07 |  | 0.06 | 0.07 | 0.06 | 0.07 |  |
|  | **2 copies** | 0.83 |  | 0.88 | 0.82 | 0.85 | 0.78 |  |
|  | **3 copies** | 0.09 |  | 0.05 | 0.10 | 0.08 | 0.14 |  |
|  | **4 copies** | 0.01 |  | 0.00 | 0.01 | 0.02 | 0.01 | 0.315 |
| **CNR2** |  |  |  |  |  |  |  |  |
| ***FCGR3A + FCGR2C*** | **1 copy** | 0.01 |  | 0.02 | 0.01 | 0.01 | 0.00 |  |
|  | **2 copies** | 0.94 |  | 0.96 | 0.94 | 0.94 | 0.93 |  |
|  | **3 copies** | 0.04 |  | 0.02 | 0.04 | 0.06 | 0.06 |  |
|  | **4 copies** | 0.00 |  | 0.00 | 0.00 | 0.00 | 0.01 | 0.304 |
| **CNR3** |  |  |  |  |  |  |  |  |
| ***FCGR3A + FCGR2C*** | **1 copy** | 0.00 |  | 0.00 | 0.00 | 0.00 | 0.00 |  |
|  | **2 copies** | 1.00 |  | 0.99 | 1.00 | 1.00 | 0.99 |  |
|  | **3 copies** | 0.00 |  | 0.01 | 0.00 | 0.00 | 0.01 | 0.068 |
| ***FCGR2A*** |  |  |  |  |  |  |  |  |
|  | **131 H** | 0.54 |  | 0.54 | 0.56 | 0.54 | 0.48 |  |
|  | **131 R** | 0.46 |  | 0.46 | 0.44 | 0.46 | 0.52 | 0.263 |
|  | **27 Q** | 0.88 |  | 0.87 | 0.88 | 0.89 | 0.91 |  |
|  | **27 W** | 0.12 |  | 0.13 | 0.12 | 0.11 | 0.09 | 0.511 |
| ***FCGR3A*** |  |  |  |  |  |  |  |  |
|  | **158 F** | 0.64 |  | 0.64 | 0.64 | 0.66 | 0.63 |  |
|  | **158 V** | 0.36 |  | 0.36 | 0.36 | 0.35 | 0.37 | 0.795 |
| ***FCGR2C*** |  |  |  |  |  |  |  |  |
|  | **Stop** | 0.84 |  | 0.87 | 0.82 | 0.85 | 0.81 |  |
|  | **Classic ORF** | 0.11 |  | 0.12 | 0.11 | 0.12 | 0.09 |  |
|  | **Nonclassic ORF** | 0.05 |  | 0.02 | 0.06 | 0.03 | 0.10 | **0.003** |
| **promoter haplotype** | **2B.1** | 0.89 |  | 0.88 | 0.89 | 0.88 | 0.93 |  |
|  | **2B.2** | 0.11 |  | 0.12 | 0.11 | 0.12 | 0.07 | 0.254 |
| ***FCGR3B*** |  |  |  |  |  |  |  |  |
|  | **NA1** | 0.35 |  | 0.33 | 0.35 | 0.38 | 0.35 |  |
|  | **NA2** | 0.62 |  | 0.66 | 0.62 | 0.60 | 0.62 |  |
|  | **SH** | 0.02 |  | 0.01 | 0.03 | 0.02 | 0.03 | 0.449 |
| ***FCGR2B*** |  |  |  |  |  |  |  |  |
|  | **232I** | 0.87 |  | 0.89 | 0.85 | 0.88 | 0.90 |  |
|  | **232T** | 0.13 |  | 0.11 | 0.15 | 0.12 | 0.10 | 0.761 |
| **promoter haplotype** | **2B.1** | 0.90 |  | 0.91 | 0.90 | 0.89 | 0.89 |  |
|  | **2B.4** | 0.10 |  | 0.09 | 0.10 | 0.11 | 0.11 | 0.569 |

African

|  |  | **All African**  **(n=508)** |  | **South African**  **(n=149)** | **Ethiopian**  **(n=142)** | **West African**  **(n=65)** | **Surinam**  **(n=78)** | **Antillean**  **(n=74)** | **Fisher’s exact** |
| --- | --- | --- | --- | --- | --- | --- | --- | --- | --- |
| **CNR1** |  |  |  |  |  |  |  |  |  |
| ***FCGR3B + FCGR2C*** | **0 copies** | 0.00 |  | 0.00 | 0.01 | 0.02 | 0.00 | 0.00 |  |
|  | **1 copy** | 0.11 |  | 0.05 | 0.15 | 0.17 | 0.14 | 0.09 |  |
|  | **2 copies** | 0.73 |  | 0.70 | 0.68 | 0.77 | 0.77 | 0.84 |  |
|  | **3 copies** | 0.14 |  | 0.23 | 0.16 | 0.05 | 0.08 | 0.07 |  |
|  | **4 copies** | 0.01 |  | 0.03 | 0.01 | 0.00 | 0.01 | 0.00 | **<0.001** |
| **CNR2** |  |  |  |  |  |  |  |  |  |
| ***FCGR3A + FCGR2C*** | **1 copy** | 0.01 |  | 0.00 | 0.00 | 0.00 | 0.03 | 0.04 |  |
|  | **2 copies** | 0.96 |  | 0.98 | 0.97 | 1.00 | 0.91 | 0.91 |  |
|  | **3 copies** | 0.03 |  | 0.02 | 0.03 | 0.00 | 0.06 | 0.05 |  |
|  | **4 copies** | 0.00 |  | 0.00 | 0.00 | 0.00 | 0.00 | 0.00 | **0.010** |
| **CNR3** |  |  |  |  |  |  |  |  |  |
| ***FCGR3A + FCGR2C*** | **1 copy** | 0.00 |  | 0.00 | 0.00 | 0.02 | 0.00 | 0.00 |  |
|  | **2 copies** | 1.00 |  | 1.00 | 1.00 | 0.97 | 1.00 | 1.00 |  |
|  | **3 copies** | 0.00 |  | 0.00 | 0.00 | 0.02 | 0.00 | 0.00 | **0.016** |
| ***FCGR2A*** |  |  |  |  |  |  |  |  |  |
|  | **131 H** | 0.44 |  | 0.39 | 0.46 | 0.42 | 0.50 | 0.45 |  |
|  | **131 R** | 0.56 |  | 0.61 | 0.54 | 0.58 | 0.50 | 0.55 | 0.175 |
|  | **27 Q** | 0.89 |  | 0.87 | 0.86 | 0.93 | 0.97 | 0.89 |  |
|  | **27 W** | 0.11 |  | 0.13 | 0.14 | 0.07 | 0.03 | 0.11 | **<0.001** |
| ***FCGR3A*** |  |  |  |  |  |  |  |  |  |
|  | **158 F** | 0.64 |  | 0.65 | 0.58 | 0.64 | 0.69 | 0.69 |  |
|  | **158 V** | 0.36 |  | 0.35 | 0.42 | 0.36 | 0.31 | 0.31 | 0.104 |
| ***FCGR2C*** |  |  |  |  |  |  |  |  |  |
|  | **Stop** | 0.90 |  | 0.88 | 0.92 | 0.93 | 0.94 | 0.88 |  |
|  | **Classic ORF** | 0.02 |  | 0.00 | 0.03 | 0.02 | 0.03 | 0.03 |  |
|  | **Nonclassic ORF** | 0.08 |  | 0.12 | 0.05 | 0.06 | 0.04 | 0.08 | **<0.001** |
| **promoter haplotype** | **2B.1** | 0.95 |  | 0.93 | 0.95 | 1.00 | 0.97 | 0.95 |  |
|  | **2B.2** | 0.05 |  | 0.07 | 0.05 | 0.00 | 0.03 | 0.05 | **0.011** |
| ***FCGR3B*** |  |  |  |  |  |  |  |  |  |
|  | **NA1** | 0.38 |  | 0.47 | 0.26 | 0.45 | 0.36 | 0.40 |  |
|  | **NA2** | 0.46 |  | 0.30 | 0.65 | 0.37 | 0.52 | 0.51 |  |
|  | **SH** | 0.15 |  | 0.23 | 0.10 | 0.18 | 0.12 | 0.09 | **<0.0001** |
| ***FCGR2B*** |  |  |  |  |  |  |  |  |  |
|  | **232I** | 0.73 |  | 0.71 | 0.77 | 0.69 | 0.67 | 0.81 |  |
|  | **232T** | 0.27 |  | 0.29 | 0.23 | 0.31 | 0.33 | 0.19 | **0.027** |
| **promoter haplotype** | **2B.1** | 0.99 |  | 1.00 | 0.98 | 1.00 | 0.98 | 0.98 |  |
|  | **2B.4** | 0.01 |  | 0.00 | 0.02 | 0.00 | 0.02 | 0.02 | **0.019** |

**Table S3**. Prevalence of CNVs (CNRs, proportion of individuals with that number of copies is shown) and SNPs (allele frequencies are shown). Overall P: Fisher's exact test for differences between populations for that variation. NL: Netherlands, UK: United Kingdom

**Table S4. Haplotypes of *FCGR2C***

|  | |  | **Europeans** | | **Chinese** | | **African** | |
| --- | --- | --- | --- | --- | --- | --- | --- | --- |
| **SNP** | |  | **allele frequency** | | **allele frequency** | | **allele frequency** | |
| p.Q57X | | p.57Q | 308/1909 | 0.16 | 4/917 | 0.00 | 101/1049 | 0.10 |
| c.798+1A>G | | c.798+1G | 247/1909 | 0.13 | 4/917 | 0.00 | 25/1049 | 0.02 |
| Linkage Disequilibrium  (diploid only) | | D' | 0.856 | | 1.00 | | 0.934 | |
|  |  | R^2 | 0.724 | | 1.00 | | 0.302 | |
| **Haplotype** | **exon3** | **intron7** | **haplotype frequency** | | **haplotype frequency** | | **haplotype frequency** | |
| 1. Classic *FCGR2C*-ORF | p.57Q | c.798+1G | 213/1909 | 0.11 | 2/917 | 0.00 | 22/1049 | 0.02 |
| 2. Nonclassic *FCGR2C*-ORF | p.57Q | c.798+1A | 99/1909 | 0.05 | 2/917 | 0.00 | 79/1049 | 0.08 |
| 3. *FCGR2C*-Stop(1) | p.57X | c.798+1A | 1565/1909 | 0.82 | 911/917 | 0.99 | 945/1049 | 0.90 |
| 4. *FCGR2C*-Stop(2) | p.57X | c.798+1G | 32/1909 | 0.02 | 2/917 | 0.00 | 3/1049 | 0.00 |

**Table S4.** Upper panel: SNP frequencies for the Q57X SNP (rs759550223) and the c.798+1A>G SNP (rs76277413) for the different populations, with strong LD between p.57Q and c.798+1G. Although MLPA cannot determine whether p.57Q and c.798+1G in a given sample are on the same chromosome, the very strong linkage suggests that these two variants are on the same chromosome if present in one individual, designated as the classic *FCGR2C*-ORF allele. In all cases in which p.57Q was accompanied by c.798+1A, this second haplotype was designated “nonclassic” *FCGR2C*-ORF. Cases with p.57X and c.798+1A or c.798+1G are designated *FCGR2C*-Stop(1) and *FCGR2C*-Stop(2), respectively. The lower panel shows the four different haplotypes that are distinguished, with the relative haplotype frequency indicated (a schematic representation of the different haplotypes is shown in Figure 1e of the manuscript).

Theoretically, misinterpretation will occur when there is a nonclassic *FCGR2C*-ORF on one chromosome and an *FCGR2C*-Stop(2) on the other chromosome. However, since the nonclassic *FCGR2C*-ORF and *FCGR2C*-Stop(2) alleles are rare in all populations, any misinterpretation about their location on one chromosome will occur in only ~0.1% (5.2% times 1.7% = 0.0884%) of European individuals, and even less in other populations.

**Table S5. Linkage Disequilibrium between CNR1 and SNPs at the *FCGR2/3*** locus.

| **European** |  | |  |  |  | | |  | | |  |
| --- | --- | --- | --- | --- | --- | --- | --- | --- | --- | --- | --- |
|  | ***FCGR2A*** | | | ***FCGR3A*** | ***FCGR2C*** | | | ***FCGR3B*** | | | ***FCGR2B*** |
|  | **H131R** | **Q27W** | | **V158F** | **Stop/ORF/NC-ORF** | | | **haplotypes** | | | **I232T** |
| **CNR1** | **f(H)** | **f(W)** | | **f(V)** | **f(Stop)** | **f(ORF)** | **f(NC)** | **f(NA1)** | **f(NA2)** | **f(SH)** | **f(T)** |
| **<2 (n=61)** | 49.2% | 13.1% | | 44.8% | NE | NE | NE | NE | NE | NE | 9.8% |
| **2 (n=768)** | 56.1% | 11.6% | | 34.5% | 86.5% | 11.7% | 1.8% | 34.1% | 65.5% | 0.4% | 12.7% |
| **>2 (n=90)** | 44.6% | 13.0% | | 39.2% | 69.4% | 8.0% | 22.6% | 41.7% | 44.2% | 14.1% | 6.0% |
| **P value** | **<0.01** | ns | | **<0.05** | **<0.001** | ns | **<0.001** | **<0.05** | **<0.001** | **<0.001** | **<0.05** |
|  |  |  | |  |  |  |  |  |  |  |  |
| **African** |  |  | |  |  |  |  |  |  |  |  |
|  | **H131R** | **Q27W** | | **V158F** | **Stop/ORF/NC-ORF** | | | **haplotypes** | | | **I232T** |
| **CNR1** | **f(H)** | **f(W)** | | **f(V)** | **f(Stop)** | **f(ORF)** | **f(NC)** | **f(NA1)** | **f(NA2)** | **f(SH)** | **f(T)** |
| **<2 (n=59)** | 45.8% | 15.3% | | 43.0% | NE | NE | NE | NE | NE | NE | 23.7% |
| **2 (n=372)** | 42.5% | 7.7% | | 32.4% | 92.7% | 2.3% | 5.1% | 37.0% | 48.4% | 14.7% | 28.1% |
| **>2 (n=77)** | 48.1% | 21.4% | | 46.1% | 82.7% | 0.8% | 16.5% | 39.2% | 41.8% | 19.0% | 17.6% |
| **P value** | ns | **<0.001** | | **<0.01** | **<0.001** | ns | **<0.001** | ns | ns | ns | **<0.05** |
|  |  |  | |  |  |  |  |  |  |  |  |
| **Chinese** |  |  | |  |  |  |  |  |  |  |  |
|  | **H131R** | **Q27W** | | **V158F** | **Stop/ORF/NC-ORF** | | | **haplotypes** | | | **I232T** |
| **CNR1** | **f(H)** | **f(W)** | | **f(V)** | **f(Stop)** | **f(ORF)** | **f(NC)** | **f(NA1)** | **f(NA2)** | **f(SH)** | **f(T)** |
| **<2 (n=40)** | 78.8% | 0.0% | | 32.1% | NE | NE | NE | NE | NE | NE | 32.5% |
| **2 (n=311)** | 68.6% | 0.3% | | 40.0% | 99.7% | 0.3% | 0.0% | 63.7% | 36.3% | 0.0% | 27.0% |
| **>2 (n=77)** | 55.2% | 1.3% | | 23.4% | 99.2% | 0.0% | 0.8% | 56.5% | 43.1% | 0.4% | 16.2% |
| **P value** | **<0.001** | ns | | **<0.001** | ns | ns | ns | ns | ns | ns | **<0.001** |

**Table S5**. Linkage Disequilibrium between CNR1 and SNPs at the *FCGR2/3* locus. Fisher's exact test is shown for all combinations, ns; not significant (P>0.05), NE; not estimated, these numbers are not shown because they involve SNPs that are deleted from the chromosome that shows the deletion of this CNR, and therefore theoretically cannot be in LD with that deletion. f indicates allele frequency. ORF = classic *FCGR2C*-ORF haplotype. NC = nonclassic *FCGR2C*-ORF haplotype. Polymorphic amino acids are indicated by one-letter code

**Table S6. Linkage Disequilibrium between CNR2 and SNPs at the *FCGR2/3* locus**

| **European** |  | |  |  |  | | |  | | |  |
| --- | --- | --- | --- | --- | --- | --- | --- | --- | --- | --- | --- |
|  | ***FCGR2A*** | | | ***FCGR3A*** | ***FCGR2C*** | | | ***FCGR3B*** | | | ***FCGR2B*** |
|  | **H131R** | **Q27W** | | **V158F** | **Stop/ORF/NC-ORF** | | | **haplotypes** | | | **I232T** |
| **CNR2** | **f(H)** | **f(W)** | | **f(V)** | **f(Stop)** | **f(ORF)** | **f(NC)** | **f(NA1)** | **f(NA2)** | **f(SH)** | **f(T)** |
| **<2 (n=11)** | 59.1% | 13.6% | | NE | NE | NE | NE | 31.8% | 68.2% | 0.0% | 22.7% |
| **2 (n=866)** | 54.8% | 12.2% | | 35.3% | 83.7% | 11.3% | 5.0% | 35.7% | 62.0% | 2.4% | 11.9% |
| **>2 (n=42)** | 54.2% | 12.5% | | 38.0% | 84.7% | 12.6% | 2.7% | 38.7% | 57.3% | 4.0% | 6.9% |
| **P value** | ns | ns | | ns | ns | ns | ns | ns | ns | ns | **<0.001** |
|  |  |  | |  |  |  |  |  |  |  |  |
| **African** |  |  | |  |  |  |  |  |  |  |  |
|  | **H131R** | **Q27W** | | **V158F** | **Stop/ORF/NC-ORF** | | | **haplotypes** | | | **I232T** |
| **CNR2** | **f(H)** | **f(W)** | | **f(V)** | **f(Stop)** | **f(ORF)** | **f(NC)** | **f(NA1)** | **f(NA2)** | **f(SH)** | **f(T)** |
| **<2 (n=5)** | 50.0% | 10.0% | | NE | NE | NE | NE | 50.0% | 50.0% | 0.0% | 10.0% |
| **2 (n=487)** | 43.9% | 10.8% | | 36.1% | 90.8% | 2.0% | 7.2% | 36.6% | 47.6% | 15.8% | 27.2% |
| **>2 (n=16)** | 37.5% | 6.3% | | 31.9% | 80.9% | 4.3% | 14.9% | 48.5% | 39.4% | 12.1% | 15.6% |
| **P value** | ns | ns | | ns | ns | ns | ns | ns | ns | ns | ns |
|  |  |  | |  |  |  |  |  |  |  |  |
| **Chinese** |  |  | |  |  |  |  |  |  |  |  |
|  | **H131R** | **Q27W** | | **V158F** | **Stop/ORF/NC-ORF** | | | **haplotypes** | | | **I232T** |
| **CNR2** | **f(H)** | **f(W)** | | **f(V)** | **f(Stop)** | **f(ORF)** | **f(NC)** | **f(NA1)** | **f(NA2)** | **f(SH)** | **f(T)** |
| **<2 (n=3)** | 83.3% | 0.0% | | NE | NE | NE | NE | 50.0% | 50.0% | 0.0% | 0.0% |
| **2 (n=409)** | 67.0% | 0.5% | | 36.8% | 99.5% | 0.2% | 0.2% | 62.8% | 37.1% | 0.1% | 25.7% |
| **>2 (n=16)** | 68.8% | 0.0% | | 22.9% | 100.0% | 0.0% | 0.0% | 53.1% | 46.9% | 0.0% | 28.1% |
| **P value** | ns | ns | | ns | ns | ns | ns | ns | ns | ns | ns |

**Table S6.** Linkage Disequilibrium between CNR2 and SNPs at the *FCGR2/3* locus in healthy controls of different populations. Fisher's exact test is shown for all combinations, ns; not significant (P>0.05), NE; not estimated, these numbers are not shown because they involve SNPs that are deleted from the chromosome that shows the deletion of this CNR, and therefore theoretically cannot be in LD with that deletion. f indicates allele frequency. ORF = classic *FCGR2C*-ORF haplotype. NC = nonclassic *FCGR2C*-ORF haplotype. Polymorphic amino acids are indicated by one-letter code

**Table S7. Clinic**al data of the patients with KD

|  | **All patients**  **with KD**  **(n=993)** | **Case-control**  **study**  **(n=405)** | **Family-based study**  **(n=588)** |
| --- | --- | --- | --- |
| **Male gender** | 611 (61.5) | 256 (63.2) | 355 (60.4) |
| **IVIg response** |  |  |  |
| **Not treated with IVIg** | 43 (4.3) | 27 (6.6) | 16 (2.7) |
| **Treated <10 days** | 750 (75.5) | 279 (68.9) | 471 (80.1) |
| **Responder** | 582 (58.6) | 221 (54.6) | 361 (61.4) |
| **Non-responder** | 168 (16.9) | 58 (14.3) | 110 (18.7) |
| **Treated >10 days** | 107 (10.8) | 58 (14.3) | 49 (8.3) |
| **Data missing** | 93 (9.4) | 41 (10.1) | 52 (8.8) |
| **Coronary outcome** |  |  |  |
| **No CAA** | 676 (62.4) | 292 (72.1) | 384 (65.3) |
| **CAA** | 255 (25.7) | 90 (22.2) | 165 (28.0) |
| **Data missing** | 62 (6.2) | 23 (5.7) | 39 (6.6) |

**Table S7.** Clinical data of the patients with KD. Clinical data were available for 993 (96.6%) out of a total of 1028 patients with KD.

Data are presented as n (%). The relatively high frequency of CAL and poor IVIg response within our cohorts are not considered representative of the true frequencies within the general populations of KD patients and are probably biased by referral to tertiary care hospitals and increased participation rates of patients with a more severe outcome.

Abbreviations: CAA=coronary artery aneurysm; IVIg=intravenous immunoglobulin; KD=Kawasaki disease.

**Table S8. Probes in gene expression microarray**

| **Gene** | **Probe sequence** | **Location** | **Specificity** |
| --- | --- | --- | --- |
| FCGR1A | 5’-GCGAGGCTGCCACAGAGGATGGA  AATGTCCTTAAGCGCAGCCCTGAGTTG-3’ | Exon5 of FCGR1A (perfect match) | May also bind pseudogenes FCGR1C (perfect match) and FCGR1B (1 mismatch) |
| FCGR2A | 5’-CTGGACGTTGGCACAGTGCTGGGATGA  CTATGGAGACCCAAATGTCTCAG-3’ | Exon1 of FCGR2A  (perfect match) | Highly specific |
| FCGR2B | 5’–GGAAGATCTGGTATTTCCTGGCCTAAAT  TCCCCTTGGGGAGGACAGGGAG–3’ | 3’ UTR of FCGR2B  (perfect match) | Highly specific |
| FCGR3A | 5’– TTCCTTCCTGGTCCTGTTCTATGGTGGGGCT  CCCTTGCCAGACTTCAGAC –3’ | 3’ UTR of FCGR3A  (perfect match) | Similar sequence in FCGR3B has 5 mismatches |
| FCGR3B | 5’–ACGCTGTGAAACTTTCAAATCCTTCTTCA  GTCAGTTCCAATGAGGTGGGG–3’ | 3’ UTR of FCGR3B  (1 mismatch) | Similar sequence in FCGR3A has 5 mismatches |

**Table S9. Primers used in RT-qPCR analysis**

|  | **Sequence** | **Product size** | **Average Slope** | **Efficiency** |
| --- | --- | --- | --- | --- |
| **GUS** | | | | |
| Forward primer | 5’-GAAAATATGTGGTTGGAGAGCTCATT-3’ | 100 bp | -3.59 | 1.899 |
| Reverse primer | 5’-CCGAGTGAAGATCCCCTTTTTA-3’ |
| **GAPDH** | | | | |
| Forward primer | 5’–TGCACCACCAACTGCTTAGC–3’ | 87 bp | -3.49 | 1.936 |
| Reverse primer | 5’–GGCATGGACTGTGGTCATGAG–3’ |
| **FCGR2A** | | | | |
| Forward primer | 5’-ATCATTGTGGCTGTGGTCATTGC-3’ | 275 bp | -3.82 | 1.829 |
| Reverse primer | 5’-TCAGGTAGATGTTTTTATCATCG-3’ |
| **FCGR2B2** | | | | |
| Forward primer | 5’-GGAAAAAGCGCATTTGAGCCAATC-3’ | 193 bp | -3.70 | 1.863 |
| Reverse primer | 5’-GGAAATACGAGATCTTCCCTCTCTG-3’ |
| **FCGR2C** | | | | |
| Forward primer | 5’-ATCATTGTGGCTGTGGTCACTGG-3’ | 160 bp | -4.10 | 1.753 |
| Reverse primer | 5’-CTTTCTGATGGCAATCATTTGACG-3’ |
| **FCGR3A** | | | | |
| Forward primer | 5’-CACATATTTACAGAATGGCACAGG-3’ | 170 bp | -3.73 | 1.855 |
| Reverse primer | 5’-ACACTGCCAAACCTTGAGTGATGG-3’ * |
| **FCGR3B** | | | | |
| Forward primer | 5’-CACATATTTACAGAATGGCAAGGA-3’ | 170 bp | -3.72 | 1.859 |
| Reverse primer | 5’-ACACTGCCAAACCTTGAGTGATGG-3’ * |
| * The same reverse primer is used for FCGR3A and FCGR3B, specificity derives from the forward primer | | | | |

**Figure S1. Gating strategy for blood leukocytes**

**A**

**
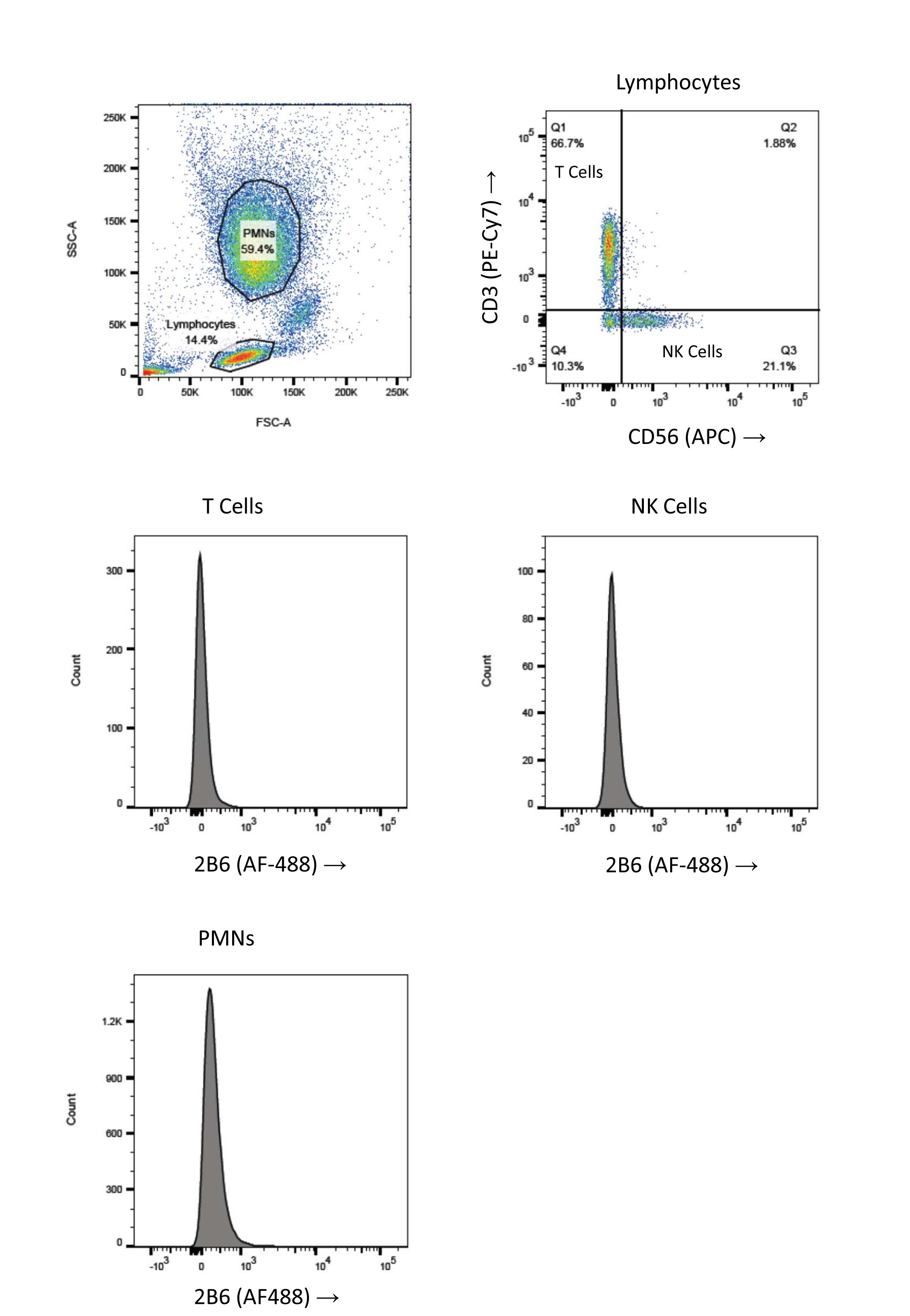
**

**B**

**
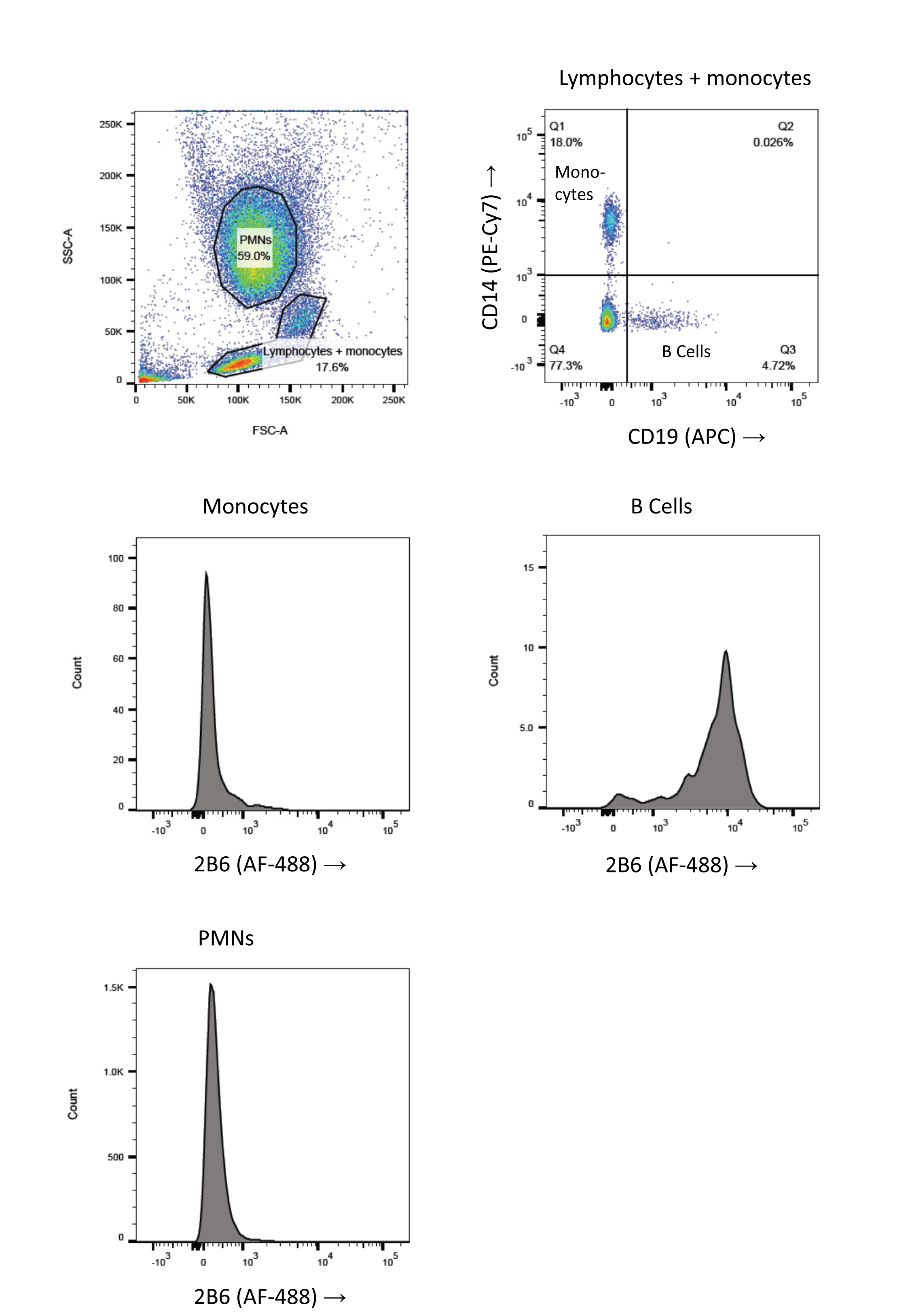
**

**Figure S1. Gating strategy for blood leukocytes**

**A.** Gating strategy for T cells, NK cells and neutrophils. Cells were stained with a mixture of antibodies staining for CD3, CD56 and the FcγR of interest, in this case FcγRIIb and FcγRIIc stained by MoAb 2B6. Lymphocytes were gated away from monocytes and polymorphonuclear cells (PMNs) on forward/side scatter pattern. Lymphocytes were subsequently gated into CD3^pos^CD56^neg^ cells (T cells) and CD3^neg^CD56^pos^ cells (NK cells). Neutrophils were defined on forward/side scatter pattern alone, including all the cells in the PMN gate. A small percentage of CD3^pos^CD56^pos^ cells (largely representing NKT cells) was usually present but was not analyzed because of low numbers.

**B.** Gating strategy for monocytes, B cells and neutrophils. Cells were stained with a mixture of antibodies staining for CD14, CD19 and the FcγR of interest, in this case FcγRIIb and FcγRIIc stained by MoAb 2B6. Lymphocytes and monocytes together were gated away from PMNs on forward/side scatter pattern. The combined gate containing lymphocytes and monocytes was subsequently gated into CD14^pos^CD19^neg^ cells (monocytes) and CD14^neg^CD19^pos^ cells (B cells). Neutrophils were defined on forward/side scatter pattern alone, including all the cells in the PMN gate.

**Figure S2. Linkage disequilibrium at the *FCGR2/3* locus – D’**


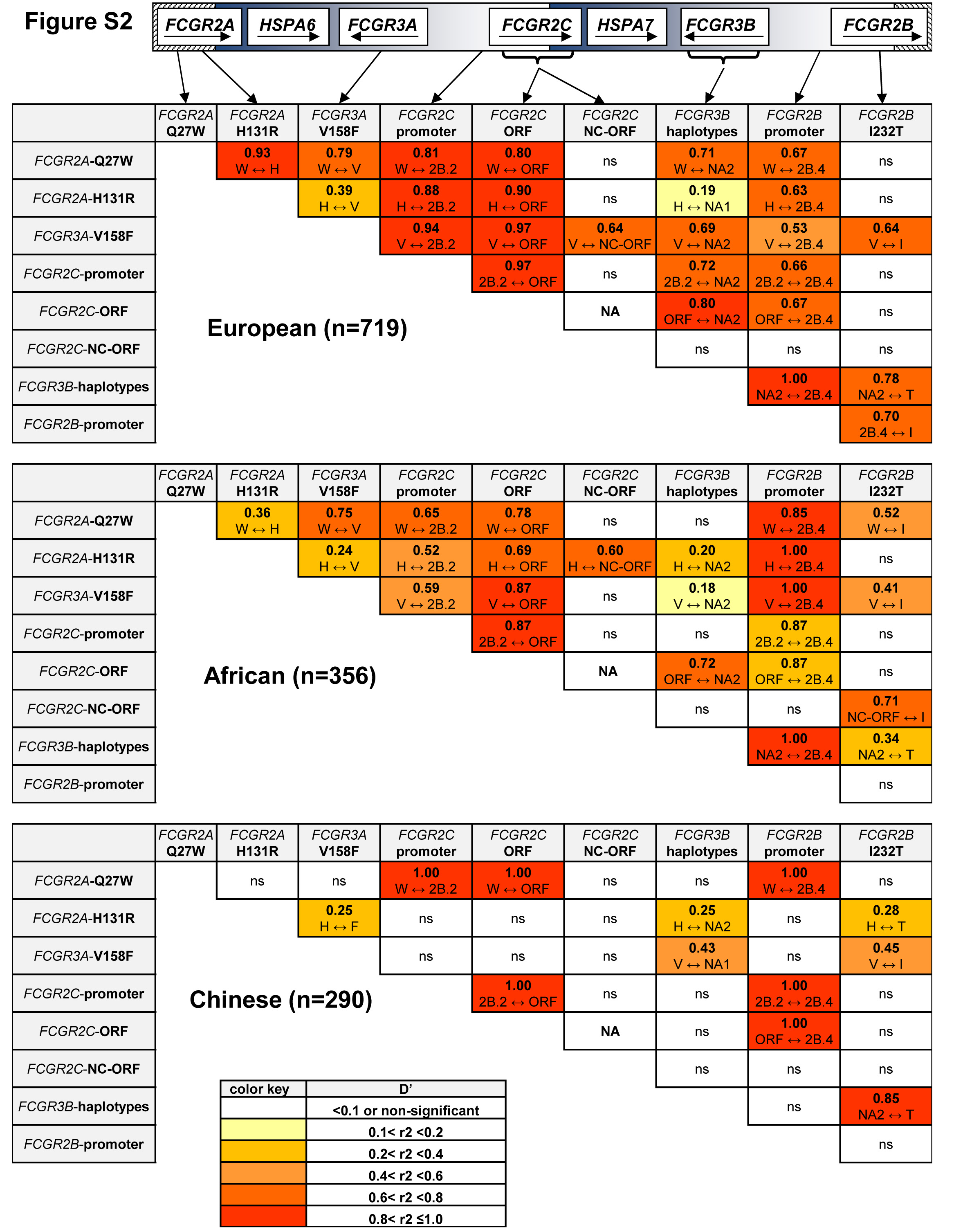


**Figure S2.** Linkage Disequilibrium for SNPs and haplotypes in individuals without CNV. D’ is shown for all combinations, which variant is linked to which variant is shown underneath. ns = non-significant (P > 0.05). *FCGR2C*-ORF = classic *FCGR2C*-ORF haplotype vs all other *FCGR2C* haplotypes. *FCGR2C*-NC-ORF = nonclassic *FCGR2C*-ORF haplotype vs all other *FCGR2C* haplotypes*.* NA: not available, because the classic *FCGR2C*-ORF haplotype and nonclassic *FCGR2C*-ORF haplotype are mutually exclusive. Polymorphic amino acids are indicated by one-letter code.

**Figure S3. Linkage Disequilibrium at the *FCGR2/3* locus in European parents of KD Family-based association test.**

**
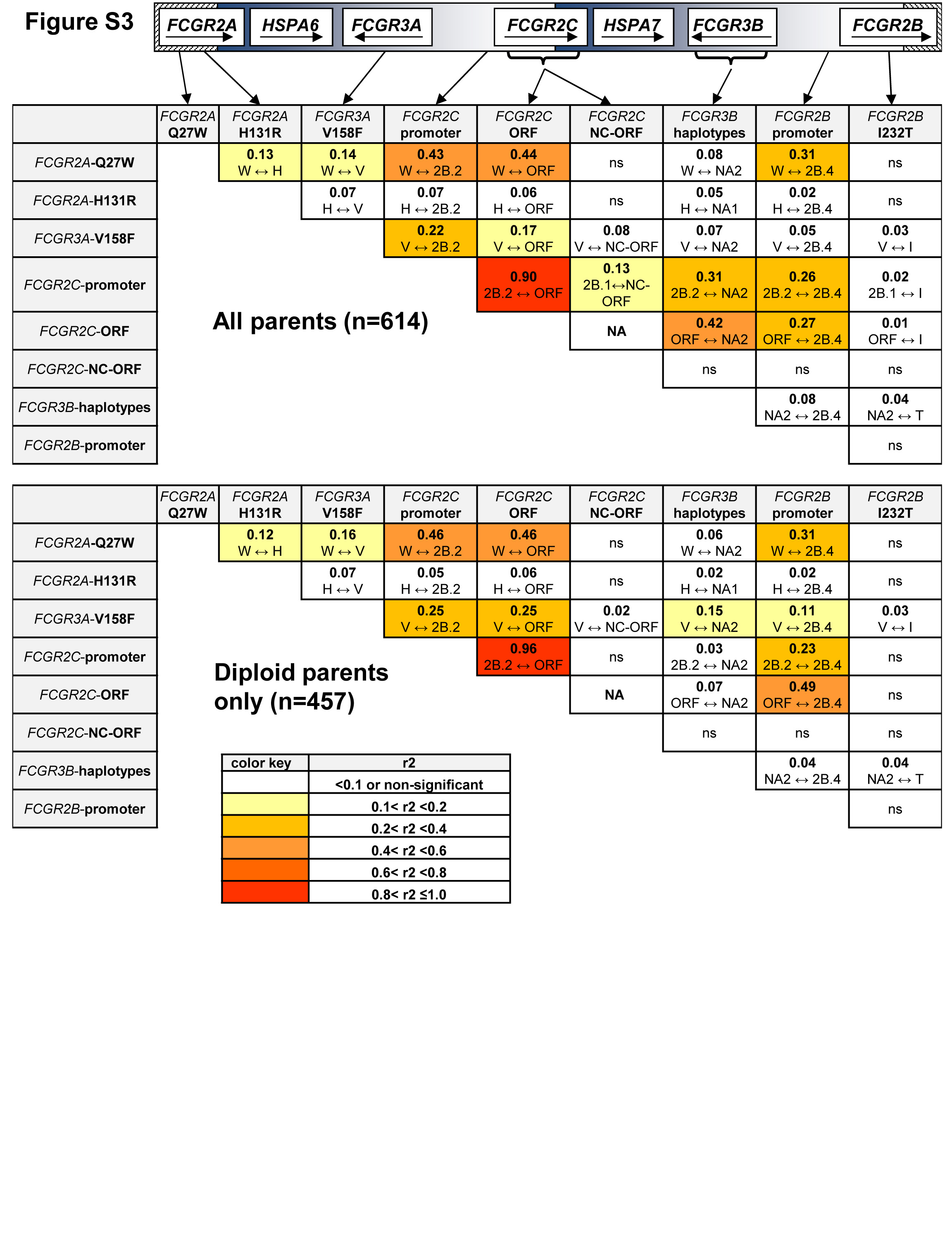
**

**Figure S3.** Linkage Disequilibrium for SNPs and haplotypes in parents of the KD Family-based association test. Results are shown for all parents (upper panel, n=614) or for only the parents with two copies of each CNR (diploid only, lower panel, n=457). r2 is shown for all combinations, which variant is linked to which variant is shown in brackets. ns = non-significant (P > 0.05). *FCGR2C*-ORF = classic *FCGR2C*-ORF haplotype vs all other *FCGR2C* haplotypes. *FCGR2C*-NC-ORF = nonclassic *FCGR2C*-ORF haplotype vs all other *FCGR2C* haplotypes*.* NA: not available, because the classic *FCGR2C*-ORF haplotype and nonclassic *FCGR2C*-ORF haplotype are mutually exclusive. Polymorphic amino acids are indicated by one-letter code.

**Figure S4. Expression levels of FcγRIIa are not influenced by the *FCGR2A*-Q27W polymorphism**


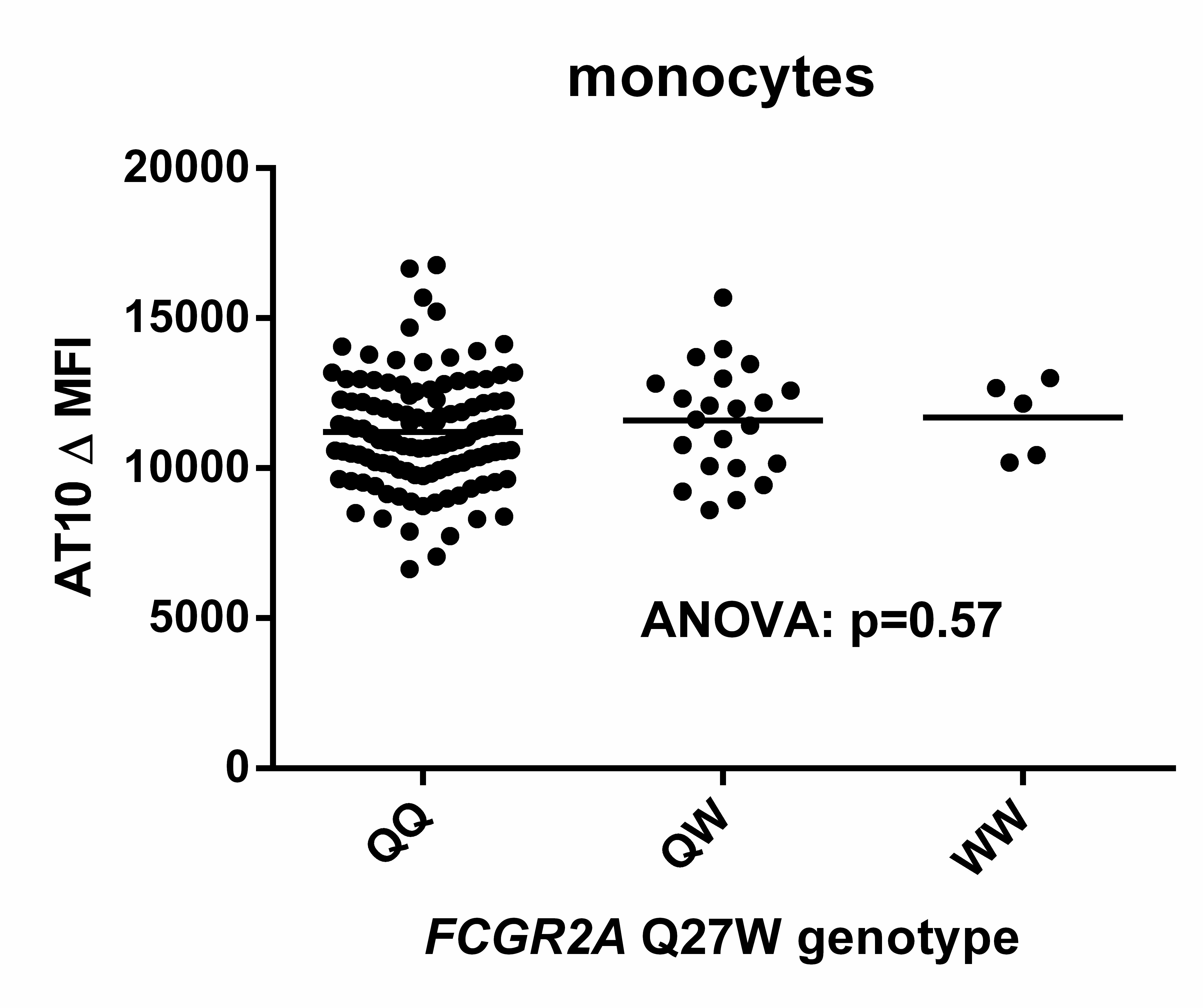

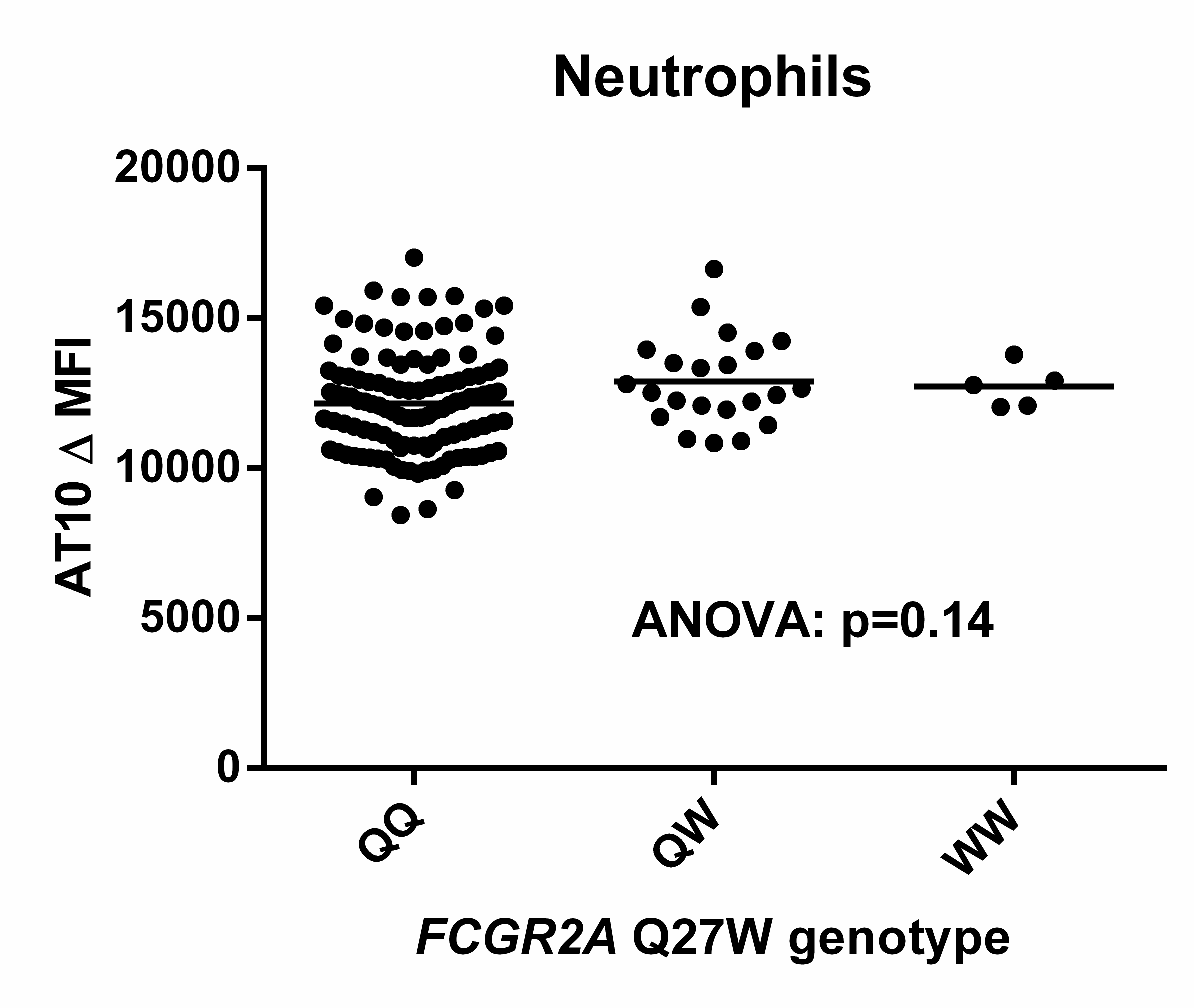


**Figure S4.** Summary of AT10 staining, corrected for isotype control, on human monocytes (left) and neutrophils (right) of donors genotyped for the *FCGR2A*-Q27W polymorphism. QQ: n=114, QW: n=22, WW: n=5. Some individuals were analyzed more than once at different time points with similar results, means are shown for these individuals. Statistical testing with one-way ANOVA, results are shown in figure.

**Supplemental Methods**

**MLPA**

Genomic DNA was isolated from whole blood with the QIAamp® Blood Mini kit (Qiagen, Hilden, Germany), or from saliva with the Oragene®DNA self-collection kit (DNA Genotek, Ontario, Canada) according to the manufacturer’s instructions.

CNVs and SNPs in the low-affinity FCGR genes FCGR2A, FCGR2B, FCGR2C, FCGR3A, and FCGR3B were determined with an FCGR-specific multiplex ligation-dependent probe amplification (MLPA) assay (MRC-Holland, Amsterdam, The Netherlands). The MLPA assay was performed according to the manufacturer’s protocol, essentially as described previously^8, 11^. Several DNA samples of individuals well-typed previously for all SNPs and CNVs were taken along in every experiment as a reference. Results were analyzed with either the program Genemarker version 1.40 (Soft Genetics LLC, State College, PA) or Coffalyser.Net (MRC-Holland, Amsterdam, The Netherlands), both software programs giving identical results. The FCGR MLPA included gene-specific probes to determine the CNV of the genes. It also included probes to detect the following SNPs: rs9427397 and rs9427398 (combined rs201218628, FCGR2A c.184C>T and c.185A>G, together forming *FCGR2A*-Q27W), rs1801274 (FCGR2A c497A>G, H131R), rs1050501 (FCGR2B c.695T>C, I232T), rs396991 (FCGR3A c.526G>T, V158F). Sequences of the probes are available on the website of MRC Holland ([http://www.mlpa.com](http://www.mlpa.com/) → products → immunological → P110 FCGR mix 1 and P111 FCGR mix 2).

In the case of *FCGR3A*-V158F, one probe was specific for 158F variant, whereas the other probe detected both *FCGR3A*-158V and *FCGR3B*^8^. The number *FCGR3A*-158V copies was calculated by subtracting the number of copies of *FCGR3B* (as assessed by gene-specific probes) from the total number of copies determined by the *FCGR3A*-158V/*FCGR3B* probe.

**Construction of haplotypes from MLPA data**

FCGR3B haplotypes (NA1/NA2/SH, also known as HNA1a/HNA1b/HNA1c, respectively, determined by 6 SNPs, rs numbers see Table S2) were constructed as follows: one probe detected the first two SNPs of the NA2 (and SH) haplotype (c.108C (rs200688856) and c.114T (rs527909462)), whereas the probe detecting the other variants (c.108G and c.114C) binds to both *FCGR3B*-NA1 and *FCGR3A*. The number of copies of *FCGR3B*-NA1 was calculated by subtracting the number of copies of *FCGR3A* (as assessed by gene-specific probes) from the total copy number determined by the *FCGR3B*-NA1/*FCGR3A* probe. A separate probe was developed specifically for SH (c.233A, rs5030738). Since SH is identical to NA2 on the other SNPs determining *FCGR3B* haplotypes^12^, for every SH, one NA2 as found by the *FCGR3B*-NA2 probe was subtracted.

*FCGR2C* haplotypes were constructed as follows: a probe specific for the stop codon in exon3 of the FCGR2C gene, (c.169T, p.57X, rs759550223) and a nonspecific FCGR2B/C probe to detect the open reading frame (ORF) in exon3 (c.169C, p.57Q in *FCGR2B* and *FCGR2C*), the number of c.169C copies in *FCGR2C* was determined by subtracting two copies of *FCGR2B* (which never shows CNV^11, 11^) from the total number of copies determined by the *FCGR2B/2C* ORF probe. Probes were also included for the splice site mutation at the border of exon7/intron7 in *FCGR2C* (rs76277413 c.798 +1 A>G), leading to four different haplotypes: *FCGR2C­­*-Stop(1) (p.57X and c.798+1A), *FCGR2C*-Stop(2) (p.57X and c.798+1G), classic *FCGR2C-*ORF (p.57Q and c.798+1G) and non-classic *FCGR2C*-ORF (p.57Q and c.798+1A) (See also Figure S1).

The assay also contained nonspecific probes for the promoter regions of *FCGR2B* and *FCGR2C*; FCGR2B/C –386C>G (rs143796418 in *FCGR2B*, rs149754834 in *FCGR2C*) and FCGR2B/C –120A> T (rs780467580 in *FCGR2B*, rs34701572 in *FCGR2C*). The MLPA probes for FCGR2B/C –386C / G and FCGR2B/C –120A / T are not specific for *FCGR2B* or *FCGR2C* as a result of the complete sequence homology surrounding these SNPs. For each individual, the promoter haplotypes were first constructed as follows: any C at -386 accompanied by an A at -120 was designated as a 2B.4. Any additional C at -386 that was not accompanied by an A at -120 was designated 2B.2. All remaining promoter haplotypes (G at -386 with a T at -120) were designated 2B.1. Next, the observed haplotypes of a given sample were allocated to *FCGR2B* and *FCGR2C*. We and others have previously shown that the 2B.4 haplotype occurs almost exclusively in *FCGR2B*, whereas the 2B.2 haplotype occurs predominantly in *FCGR2C*^2, 4, 8, 13^. Recently, we have shown that the rare individuals that have a 2B.2 in *FCGR2C*, also have a deletion of CNR1, and in these cases the 2B.2 promoter was not associated with the classic *FCGR2C*-ORF haplotype^4^. Because of these previous observations, we allocated all 2B.4 haplotypes as detected by MLPA to *FCGR2B*. All 2B.2 haplotypes detected by MLPA were allocated in *FCGR2C*, except in rare cases where the 2B.2 was not associated with a classic *FCGR2C*-ORF and there was a deletion of CNR1, in which case they were allocated to *FCGR2B*. Finally, the 2B.1 haplotypes were allocated to *FCGR2B* and *FCGR2C*, taking into account the CNV of *FCGR2C* and the 2B.2 and 2B.4 haplotypes that had already been allocated. This standard allocation strategy could be performed in 99.9% of the cases. In some rare cases, this standard allocation could not be performed because it did not fit with a normal transmission of the haplotypes in parent-offspring trios. This occurred in only 6/4092 samples (0.1%) in which a 2B.4 had to be allocated to *FCGR2C*, and in 6 other cases (0.1%) in which a 2B.2 had to be allocated to *FCGR2B* even though there was no deletion of CNR1 and no classic *FCGR2C*-ORF haplotype.

## Flow cytometry

Whole blood leukocytes were isolated from heparin blood by lysis of red blood cells with an isotonic ammonium chloride buffer. The following mAbs were used to detect leukocyte subsets in flow cytometry: anti-CD3-PE-Cy7 clone SK7 (T cells), anti-CD14-PE-Cy7 clone M5E2 (monocytes), anti-CD19-APC clone HIB19 (B cells) and anti-CD56-APC clone B159 (NK cells), all from BD Pharmingen, San Diego, CA. Gating strategy is shown in Figure S1. FcγRIIb/IIc expression was measured with anti-FcγRIIb/c clone 2B6, Alexa Fluor 488 labeled (a generous gift from MacroGenics, Rockville, MD). FcγRIIa expression was measured with anti-FcγRII clone AT10, FITC labeled (AbD Serotec, Oxford, UK). Cells were analyzed on a FACS CANTO II machine (BD). Because FcγRIIb is also stained by 2B6, only cells that do not express FcγRIIb can be easily analyzed for FcγRIIc expression. Therefore, individuals with a deletion of CNR1 were excluded from the analysis of NK cells, and individuals with a 2B.4 promoter haplotype in *FCGR2B* were excluded from the analysis of monocytes and neutrophils, because these variants result in ectopic expression of FcγRIIb on NK cells^7^, or myeloid cells^4^, respectively**.**

**Gene expression microarray**

Gene expression data from 171 KD patients were used from a previous study by Hoang *et al.*^14^, with an additional 40 samples gathered in the same way. For all 171 patients, there was a sample in the acute phase of KD (median illness day 6 (IQR(25%-75%) 5-8.5)) and a sample in the convalescent phase after KD post-IVIG treatment, in which the acute illness had resolved (median illness day 50 (IQR(25%-75%) 40-66)). This microarray included probes with specificity for FCGR1A, FCGR2A, FCGR2B, FCGR3A and FCGR3B, but no probe for FCGR2C. Probe sequences are summarized in Table S8. Z scores were calculated as described before^14^, in brief, the Z score is the result of the signal intensity of the gene of interest corrected for the signal intensity of all genes on the microarray.

**RT-qPCR**

The same patient RNA samples as used for the gene expression microarray described above^14^ were used for this analysis. RNA was extracted from whole blood collected directly into PAXgene RNA tubes (Qiagen, Sussex, UK) as described before^14^. From the samples of which sufficient RNA was still present, RNA was converted to cDNA with the SuperScript ® III First-Strand Synthesis System for RT-PCR (Invitrogen, Waltham, Massachusetts, USA). In total, cDNA was constructed from 136 patients with acute KD and 125 patients with convalescent KD, with 118 paired samples in which cDNA was available from the same patient in both the acute and convalescent phase.

RT-qPCR on the cDNA was performed on a LightCycler machine. Expression was measured for FCGR2A, FCGR2B2, FCGR2C, FCGR3A, FCGR3B, and housekeeping genes GAPDH and GUS, using the primers in Table S9.

cDNA from whole blood leukocytes from two individuals was used to construct a standard curve for each of the analyzed transcripts, with serial 10-fold dilutions of this cDNA quantified with the method described in Technical Note No. LC 13/2001 (Roche Applied Science). The slope of the standard curves was determined by a linear regression curve as calculated by the LightCycler Software. Slopes from the standard curves from 2 individuals were averaged and used to calculate efficiencies for each of the transcripts used, according to the formula E_T_ = 10^(-1/slope)^, where E_T_ is efficiency of the target gene. Calculated efficiencies are shown in Table S9. Using this efficiency, expression of each transcript in all samples was compared to the same transcript in the reference sample, which was a randomly chosen sample from the convalescent KD group. This comparison was calculated with the formula E_T_CpT(Reference sample) – CpT(Sample of interest), where CpT indicates Crossing point of the target gene, as determined by LightCycler. Consequently, the values obtained were corrected for cDNA input by dividing the values obtained for a given transcript of interest by the value of the geometric mean of housekeeping genes GAPDH and GUS of that sample, following the MIQE guidelines^15^, resulting in a relative expression of that sample, as compared to the reference sample. This relative expression is reported in Figure 4 (including the reference sample, which has a relative expression of 1 by definition).

Since there was a difference in average cell percentages between acute and convalescent samples^14^, and some of the transcripts are exclusively expressed in certain cell types^16^, we attempted to make a correction for percentages of the white blood cells, by dividing the relative expression by the percentage of the cell type(s) that exclusively express the transcript of interest. For 100 of the paired samples, complete blood counts including white blood cell differential percentages (polymorphonuclear granulocytes, band granulocytes, lymphocytes, atypical lymphocytes, monocytes, eosinophils and basophils, with the percentage of neutrophils defined as the sum of the percentages of both polymorphonuclear and band granulocytes) were available from the time of the blood collection for RNA analysis. FCGR2A was corrected for the sum of neutrophil and monocyte percentages, since these cell types uniformly express FCGR2A, whereas lymphocytes do not^16^. FCGR2B2 is the FCGR2B transcript preferentially expressed by myeloid cells, but is also present in B cells, which precluded proper correction for this transcript. FCGR2C was corrected for the percentage of monocytes only, since these cells show the highest expression of FcγRIIc (Figure 2), whereas neutrophils express only very low levels (Figure 2), and only the minor fraction of NK cells in the lymphocytes fraction expresses FcγRIIc. NK cell numbers were not determined at the time of blood collection. FCGR3A is expressed on a fraction of monocytes and NK cells, rendering correction also impossible. Finally, FCGR3B was corrected for the percentage of neutrophils, which constitutively express this receptor at high levels^16^.

Genotype of *FCGR* genes determined by MLPA was available in 135 of the 136 patients which were measured in the acute phase. Variants known to influence expression were compared in these 135 patients, in this case using values uncorrected for cell types. A similar analysis in 124 convalescent patients yielded similar results (data not shown).

**Supplemental References**

1. Bruhns P, Iannascoli B, England P, Mancardi DA, Fernandez N, Jorieux S, and Daeron M (2009) Specificity and affinity of human Fcgamma receptors and their polymorphic variants for human IgG subclasses. Blood 113 (16):3716-3725

2. Su K, Wu J, Edberg JC, Li X, Ferguson P, Cooper GS, Langefeld CD, and Kimberly RP (2004) A promoter haplotype of the immunoreceptor tyrosine-based inhibitory motif-bearing FcgammaRIIb alters receptor expression and associates with autoimmunity. I. Regulatory FCGR2B polymorphisms and their association with systemic lupus erythematosus. J Immunol 172 (11):7186-7191

3. Blank MC, Stefanescu RN, Masuda E, Marti F, King PD, Redecha PB, Wurzburger RJ, Peterson MG, Tanaka S, and Pricop L (2005) Decreased transcription of the human FCGR2B gene mediated by the -343 G/C promoter polymorphism and association with systemic lupus erythematosus. Hum Genet 117 (2-3):220-227

4. Tsang-A-Sjoe MWP, Nagelkerke SQ, Bultink IE, Geissler J, Tanck MW, Tacke CE, Ellis JA, Zenz W, Bijl M, Berden JH, de LK, Derksen RH, Kuijpers TW, and Voskuyl AE (2016) Fc-gamma receptor polymorphisms differentially influence susceptibility to systemic lupus erythematosus and lupus nephritis. Rheumatology (Oxford) 55 (5):939-948

5. Floto RA, Clatworthy MR, Heilbronn KR, Rosner DR, MacAry PA, Rankin A, Lehner PJ, Ouwehand WH, Allen JM, Watkins NA, and Smith KG (2005) Loss of function of a lupus-associated FcgammaRIIb polymorphism through exclusion from lipid rafts. Nat Med 11 (10):1056-1058

6. Kono H, Kyogoku C, Suzuki T, Tsuchiya N, Honda H, Yamamoto K, Tokunaga K, and Honda Z (2005) FcgammaRIIB Ile232Thr transmembrane polymorphism associated with human systemic lupus erythematosus decreases affinity to lipid rafts and attenuates inhibitory effects on B cell receptor signaling. Hum Mol Genet 14 (19):2881-2892

7. van der Heijden J, Breunis WB, Geissler J, de Boer M, van den Berg TK, and Kuijpers TW (2012) Phenotypic variation in IgG receptors by nonclassical FCGR2C alleles. J Immunol 188 (3):1318-1324

8. Breunis WB, van Mirre E, Bruin M, Geissler J, de Boer M, Peters M, Roos D, de Haas M, Koene HR, and Kuijpers TW (2008) Copy number variation of the activating FCGR2C gene predisposes to idiopathic thrombocytopenic purpura. Blood 111 (3):1029-1038

9. Koene HR, Kleijer M, Algra J, Roos D, von dem Borne AE, and de Haas M (1997) Fc gammaRIIIa-158V/F polymorphism influences the binding of IgG by natural killer cell Fc gammaRIIIa, independently of the Fc gammaRIIIa-48L/R/H phenotype. Blood 90 (3):1109-1114

10. Adu B, Dodoo D, Adukpo S, Hedley PL, Arthur FK, Gerds TA, Larsen SO, Christiansen M, and Theisen M (2012) Fc Gamma Receptor IIIB (FcgammaRIIIB) Polymorphisms Are Associated with Clinical Malaria in Ghanaian Children. PLoS One 7 (9):e46197

11. Breunis WB, van Mirre E, Geissler J, Laddach N, Wolbink G, van der Schoot E, de Haas M, de Boer M, Roos D, and Kuijpers TW (2009) Copy number variation at the FCGR locus includes FCGR3A, FCGR2C and FCGR3B but not FCGR2A and FCGR2B. Hum Mutat 30 (5):E640-E650

12. Reil A, Sachs UJ, Siahanidou T, Flesch BK, and Bux J (2013) HNA-1d: a new human neutrophil antigen located on Fcgamma receptor IIIb associated with neonatal immune neutropenia. Transfusion

13. Recke A, Vidarsson G, Ludwig RJ, Freitag M, Moller S, Vonthein R, Schellenberger J, Haase O, Gorg S, Nebel A, Flachsbart F, Schreiber S, Lieb W, Glaser R, Benoit S, Sardy M, Eming R, Hertl M, Zillikens D, Konig IR, Schmidt E, and Ibrahim S (2015) Allelic and copy-number variations of FcgammaRs affect granulocyte function and susceptibility for autoimmune blistering diseases. J Autoimmun 61:36-44

14. Hoang LT, Shimizu C, Ling L, Naim AN, Khor CC, Tremoulet AH, Wright V, Levin M, Hibberd ML, and Burns JC (2014) Global gene expression profiling identifies new therapeutic targets in acute Kawasaki disease. Genome Med 6 (11):541

15. Bustin SA, Benes V, Garson JA, Hellemans J, Huggett J, Kubista M, Mueller R, Nolan T, Pfaffl MW, Shipley GL, Vandesompele J, and Wittwer CT (2009) The MIQE guidelines: minimum information for publication of quantitative real-time PCR experiments. Clin Chem 55 (4):611-622

16. Nagelkerke SQ and Kuijpers TW (2015) Immunomodulation by IVIg and the Role of Fc-Gamma Receptors: Classic Mechanisms of Action after all? Front Immunol 5:674
